# Structure–Property Relationships for the Electronic Applications of Bis-Adduct Isomers of Phenyl-C_61_ Butyric Acid Methyl Ester

**DOI:** 10.1021/acs.chemmater.3c02353

**Published:** 2023-12-28

**Authors:** Xueyan Hou, Jack F. Coker, Jun Yan, Xingyuan Shi, Mohammed Azzouzi, Flurin D. Eisner, James D. McGettrick, Sachetan M. Tuladhar, Isaac Abrahams, Jarvist M. Frost, Zhe Li, T. John S. Dennis, Jenny Nelson

**Affiliations:** † Department of Physics, Imperial College London, London SW7 2AZ, U.K.; ‡ School of Physical and Chemical Sciences, Queen Mary University of London, London E1 4NS, U.K.; § School of Science and Engineering, The Chinese University of Hong Kong, Shenzhen, Guangdong Province 518172, P. R. China; ∥ SPECIFIC, Swansea University Bay Campus, Swansea, Wales SA1 8EN, U.K.; ⊥ School of Engineering and Materials Sciences, Queen Mary University of London, London E1 4NS, U.K.; # Department of Chemistry, Xi’an Jiaotong-Liverpool University, Suzhou 215123, China

## Abstract

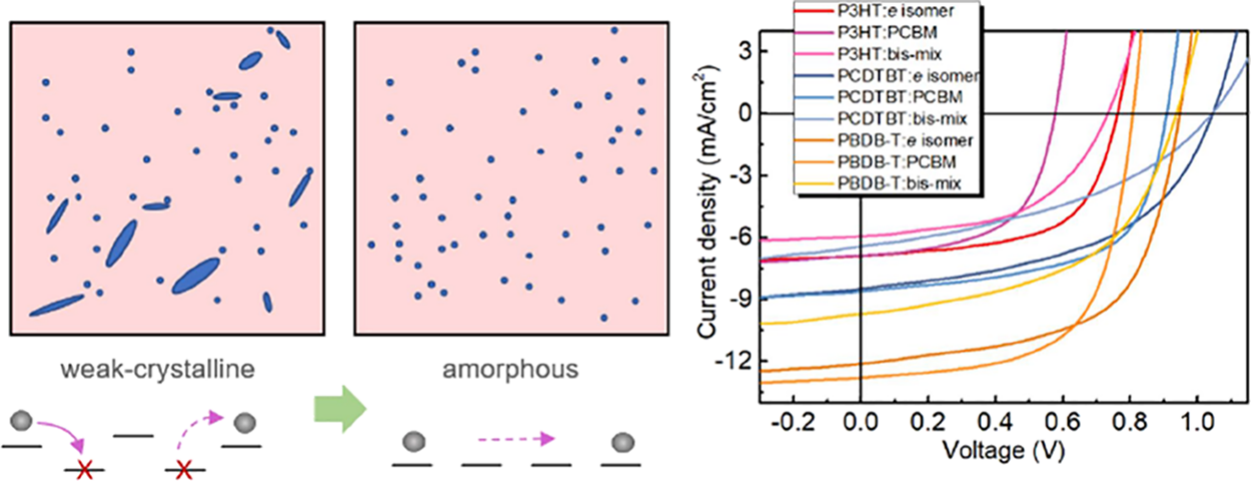

Higher adducts of a fullerene, such as the bis-adduct of PCBM (bis-PCBM), can be used to achieve shallower molecular orbital energy levels than, for example, PCBM or C_60._ Substituting the bis-adduct for the parent fullerene is useful to increase the open-circuit voltage of organic solar cells or achieve better energy alignment as electron transport layers in, for example, perovskite solar cells. However, bis-PCBM is usually synthesized as a mixture of structural isomers, which can lead to both energetic and morphological disorder, negatively affecting device performance. Here, we present a comprehensive study on the molecular properties of 19 pure bis-isomers of PCBM using a variety of characterization methods, including ultraviolet photoelectron spectroscopy, thermal gravimetric analysis, differential scanning calorimetry, single crystal structure, and (time-dependent) density functional theory calculation. We find that the lowest unoccupied molecular orbital of such bis-isomers can be tuned to be up to 170 meV shallower than PCBM and up to 100 meV shallower than the mixture of unseparated isomers. The isolated bis-isomers also show an electron mobility in organic field-effect transistors of up to 4.5 × 10^–2^ cm^2^/(V s), which is an order of magnitude higher than that of the mixture of bis-isomers. These properties enable the fabrication of the highest performing bis-PCBM organic solar cell to date, with the best device showing a power conversion efficiency of 7.2%. Interestingly, we find that the crystallinity of bis-isomers correlates negatively with electron mobility and organic solar cell device performance, which we relate to their molecular symmetry, with a lower symmetry leading to more amorphous bis-isomers, less energetic disorder, and higher dimensional electron transport. This work demonstrates the potential of side chain engineering for optimizing the performance of fullerene-based organic electronic devices.

## 1 Introduction

Fullerenes have played a central role in the past two decades in the development of organic electronic devices. Examples include acting as the active layer acceptor for organic photovoltaic devices (OPVs),^1,2^ as electron extraction and electron transport layers for perovskite solar cells,^3,4^ and as semiconductor materials for transistors and photodetectors.^5−7^ Unfunctionalized fullerenes have very low solubility, which is not compatible with solution processing of organic electronic devices, and deep-lying LUMO energies, which hinders electron injection in electronic devices. As such, fullerene derivatives of greater solubility and varying reduction potential have been synthesized by adding side chains of varying lengths or structures to the fullerene cage via cycloaddition reactions. The great majority of studies involve adjusting the phenyl butyric methyl ester side group, and the prevalent modified fullerene derivatives are mainly phenyl C_61_ and phenyl C_71_ butyric acid methyl ester (PCBM and PC_71_BM) and bis-adducts based on C_60_ or C_70_ cages. The use of multiple addends enables the tuning of fullerene redox potentials.^8^ However, multiple addends can also hinder electron transfer between fullerene cages, while the variety of isomers that is produced in the synthesis of higher adducts leads to disorder in their properties.^9^ Therefore, higher-adduct fullerene derivatives are seldom used for device applications.^8,10,11^ The limited synthetic flexibility available with single-adduct fullerenes, along with their weak optical response, has meant that the development of new fullerene materials for device applications has met a bottleneck. In the case of OPVs, alternative molecular acceptors of greater synthetic variability and more widely tunable electronic properties have taken over.^12,13^ However, the characteristics of specific isomers of fullerene derivatives rather than mixtures of chemically similar isomers are seldom studied due to the difficulty of isolating single isomers. If properly understood, the use of specific isomers could benefit electronic device performance and provide new opportunities for the application of fullerene derivatives.

Several higher-adduct derivatives of fullerenes have been synthesized. However, the use of synthesis methods that do not offer control over the attachment position of additional side chains means that they are normally used as isomeric mixtures, which are not ideal for several reasons. For example, any single isomer will possess well-defined energy levels and a structure that could, in principle, crystallize, whereas isomeric mixtures lead to both energetic and structural disorder. This disorder tends to inhibit electron transport and influence the morphology of blend films used in devices.^9,10,14^ Another problem with isomer mixtures is that the presence of even a small amount of a different isomer is known to cause disorders and traps that strongly affect device performance.^15−17^ There are several recent studies that use single isolated isomers, purified from the as-synthesized mixture, which show reduced disorder in energy levels when compared to that of the isomer mixture. These have shown that using single pure isomers can not only improve solar cell device performance, but also beneficially affect the molecular packing and crystallinity of the fullerene phase, further enhancing device thermal and chemical stability.^18−24^ Single purified bis-isomers have also been shown to work effectively as an electron transport layer for perovskite solar cells.^18^ Indeed, poor energy level alignment of transport layers with the perovskite active layer has been identified as one of the key contributors to energy losses in perovskite solar cells and in particular wide-bandgap devices.

Although isolated isomers are desirable for applications, the traditional purification method of fullerene materials, high performance liquid chromatography (HPLC), is not efficient for purifying fullerene materials that contain numerous components; it is time-consuming, considering the additional work to identify the molecular structure of each component. This limits the ability to study how multiple isomerisms affect device performance and to investigate the application of isomer-pure, high-adduct fullerenes. Using current advanced chemical engineering methods, the synthesis of single higher-adduct fullerene isomers can be achieved in conjunction with a reduction in the occurrence of different isomer species during synthesis.^25^ For example, Xiao et al. developed a prebisaddition-confined bisfunctionalization method to synthesize the target *e* configuration.^26^ Vidal et al. reported the site-selective synthesis of [70]fullerene site-isomers by controlling the solvent polarity.^27^ However, there is still a need to screen the isomers to identify which ones are good for the device performance.

Our previous work demonstrated a more efficient HPLC method to purify the well-known bis-PCBM (dimethyl 4,4′-[3′,3″-diphenyl-3*H*′,3*H*″-dicyclopropa(C_60_-I_h_)[5,6]fullerenediyl]dibutanoate) mixture, through using multiple columns to separate the isomer subgroups and to reduce the purification time. Bis-PCBM and its 19 isomers (except for chiral molecules) were all isolated with a purity of ∼99.9%.^28^ The molecular structures were previously identified therewith by the combined analysis of ^13^C NMR, UV–vis absorption spectroscopy and HPLC retention time analysis.^29^ We also found that the degradation rate of organic solar cells based on bis-PCBM isomers is correlated the LUMO energy and the crystallinity of the isomer.^23^ The bis-PCBM materials possess a higher-lying LUMO and should therefore be less stable than PCBM.

In the present work, we analyzed the energy levels, thermal properties, crystallinity, molecular packing, and optoelectronic properties, of this series of bis-PCBM isomers in order to understand the various differences in (opto)electronic performance between isomers. The best transistors and photovoltaic devices were realized by using specific isolated isomers. In addition to the benefit of single isomers in reducing energetic disorder, this work also demonstrates the important influence of molecular symmetry and molecular packing of the isomer-pure, high-adduct fullerenes on the performance of electronic devices.

## 2 Results

A mixture of bis-adducts of PCBM was synthesized according to the procedures reported by Hummelen et al. for PCBM synthesis.^30^ The raw materials, quantities, and the reaction conditions for the production of bis-adducts were based on the detailed method in ref (31). Then the as-synthesized bis-PCBM mixtures were purified using chromatography as reported in ref (28). Each of the 19 obtained isomers (purity >99.5%) was condensed to supersaturated solution and was then transferred into vacuum for slow drying for 3 days. Naming of each isomer in this paper adopts the method described in our previous work,^28^ that is, following the HPLC fraction names (see Figure S1 and Table S1).

### 2.1 Molecular Properties

**Figure 1:**
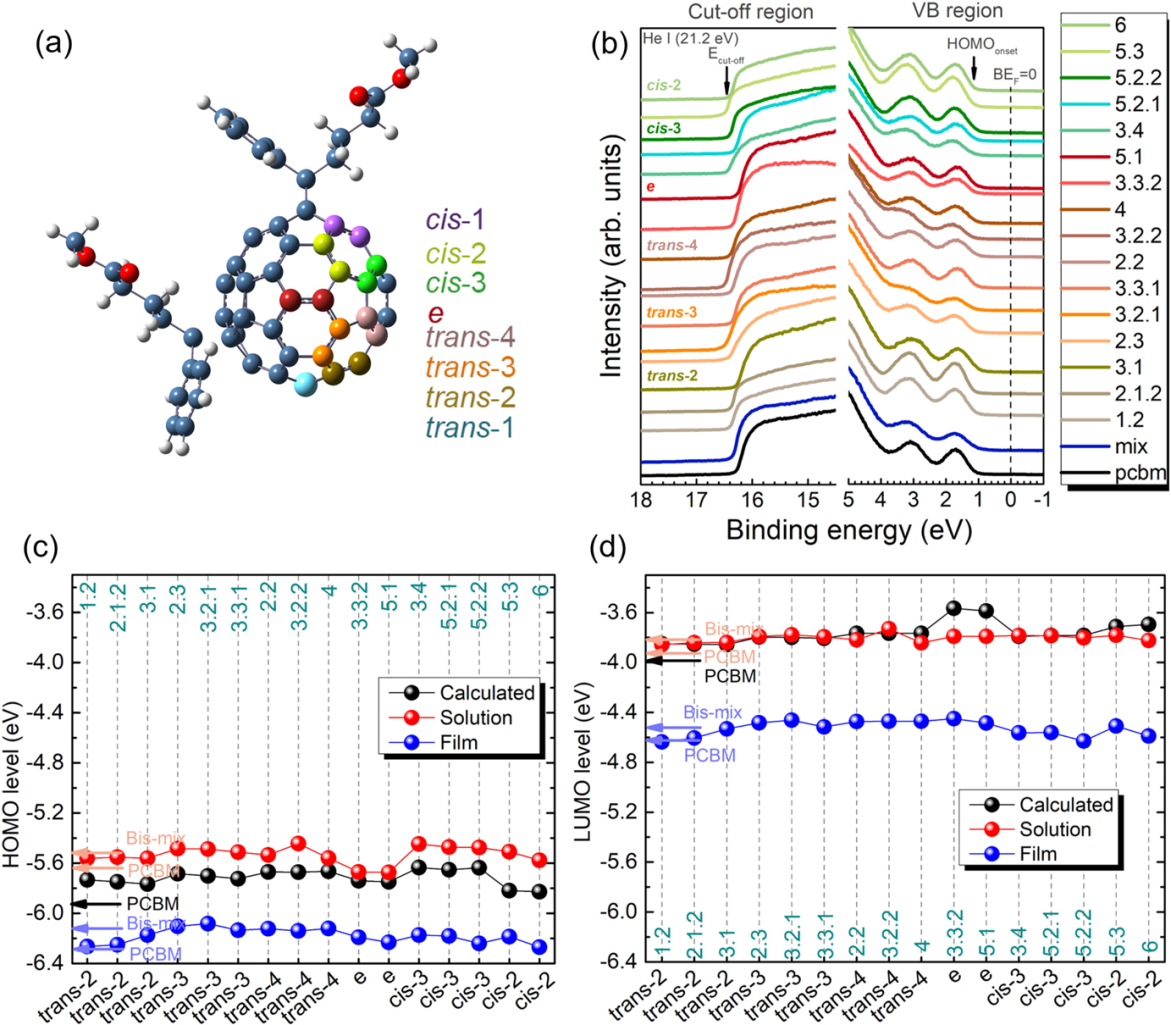
(a) Side chain distribution on the C_60_ cage varying from *trans*-1 to *cis*-1 positions. (b) Ultraviolet photoelectron spectra of different bis-PCBM isomers, which were exhibited by *trans*, *cis* and *e* groups with the cutoff region (left) and HOMO region (right). (c) HOMO energies estimated from the oxidation potential of fullerenes in solution measured by cyclic voltammetry (red) and of thin films measured using UPS (blue) in comparison with the HOMO energy calculated by DFT (black). (d) LUMO energies estimated from the reduction potential of fullerenes in solution measured by cyclic voltammetry (red) and estimated from the UPS measured HOMO energy plus the thin film measured energy gap from the UV–vis spectra (blue) in comparison with the LUMO estimated from the calculated HOMO energy plus the calculated energy of the first nondark excited state (black). HOMO and LUMO of the unseparated mixture of bis-isomers (“bis-mix”) and PCBM are indicated by colored arrows, and red, black, and blue arrows represent values from solution, calculation, and film, respectively. Note that no calculations could be performed for the bis mixture.

The eight distinct bond types and positions on the C_60_ cage for the second addend in bis-PCBM are shown at the top of Figure 1a with the first addend at the top pole position. The second addend can sit on all other C=C bonds except for the *cis*-1 ones due to steric hindrance with the first addend. To further understand how the bis-PCBM isomers differ from each other, apart from their molecular structure, we carried out a theoretical study of all 19 isomers using density functional theory (DFT) and time-dependent DFT (TD-DFT). We then characterized the isomers by experimentally probing their electronic structure, thermal properties, crystallinity, and molecular packing.

#### 2.1.1 Calculation of Electronic Properties

Figure S2 exhibits the optimized molecular geometries of the 19 bis-PCBM isomers obtained using DFT, in a vacuum, at the B3LYP/6-311G(2df, 2pd) level of theory. As in the case of the first side chain of PCBM, the addition of the second side chain does not significantly change the geometry of the C_60_ cage. For each side chain, the angle between the phenyl group and the alkyl chain is around 90° and varies little between isomers. PCBM shows *C*_s_ symmetry with the phenyl group and alkyl chain bisecting the mirror plane. The wave function density distribution (see Figure S2) shows that the electron density in the highest occupied molecular orbital (HOMO) and lowest unoccupied molecular orbital (LUMO) of the bis-PCBM isomers are primarily localized on the fullerene cages, but the density distribution pattern varies between the regioisomers. The HOMO level of the adduct was taken as the energy of the highest occupied Kohn–Sham orbital with tight convergence of the self-consistent field in DFT. Since the Kohn–Sham LUMO energy was unrealistically high, the LUMO energy was estimated by adding the HOMO energy to the transition energy of the first singlet excitation calculated using TD-DFT using the same functional and basis set.^9^ With TD-DFT, the absorption spectra were computed from the calculated excitation energies and oscillator strengths of the transitions to the first 20 excited singlet states. Since the oscillator strength is proportional to the probability of absorption or emission of electromagnetic radiation in transitions between energy levels, the HOMO–LUMO optical gap was determined as the energy of the lowest transition for which the oscillator strength exceeds 0.001. The HOMO, LUMO, and HOMO–LUMO optical gap energies are listed in Table S2. The calculated energy levels of PCBM are all deeper than the corresponding levels of the bis-PCBM isomers, which is consistent with experimental reports of shallower reduction potentials for bis-adducts.^1^ The bis-PCBM isomers show little differences in the calculated HOMO levels (−5.66 to −5.83 eV), while the LUMO levels vary more widely, from −3.03 to −3.86 eV (Table S2), a consequence of the variation in HOMO–LUMO optical gaps.

#### 2.1.2 Experimental Energy Levels

The solution state energy levels of each bis-PCBM isomer including HOMO, LUMO, and HOMO–LUMO gap were previously determined by UV–vis and cyclic voltammetry (CV).^28^ In order to measure the energy levels in the solid (film) state, we here perform UV–vis and ultraviolet photoelectron spectroscopy (UPS) measurements. The UV–vis absorption spectra of the film samples are depicted in Figure S3 and show a similar shape to the solution UV–vis spectra, with an expected red-shift in the absorption features relative to the solution state.^28^ The calculated energy gaps of the isomers are all larger than the measured gaps for both film and solution samples (from UV–vis spectra), as listed in Table S2, but all three estimates of the gap follow the same trend with chemical structure (see Figure S3g). Figure 1b depicts the UPS spectra of the secondary electron cutoff region and the HOMO onset region with the binding energy from the Fermi level of the system (BE_F_ = 0). In UPS, the work function can be obtained from the difference between the incident photon energy (HeI: 21.2 eV) and the cut-off energy (*E*_cut-off_). The HOMO level relative to the vacuum level can be derived by adding the HOMO onset energy to the work function. The cutoff energy varies from 16.30 to 16.50 eV, and the value of the HOMO energy relative to the Fermi level varies from 1.25 to 1.40 eV. The HOMO levels of bis-PCBM isomer films determined in this way are listed in Table S2 and vary between −6.10 and −6.27 eV and are all deeper than those estimated from cyclic voltammetry of the isomers in solution.^28^ LUMO levels, calculated by adding the bandgap determined by UV–vis to the HOMO level, range between −4.47 and −4.63 eV, with all but two (F1.2 and F5.2.2). For a clear comparison, the energy levels of bis-PCBM isomers from theoretical calculations and experimental measurements in solution and in film are summarized in Figure 1c (HOMO) and Figure 1d (LUMO). The HOMO and LUMO energies of the isomers in solution lie close to the theoretical estimates while lying higher than the values for films. Nevertheless, the trends in the energy level with chemical structure are quite similar. In comparison with PCBM and the isomer mixture, the highest-lying LUMO level of the isomers is ∼100 meV higher than that for the mixture and ∼170 and ∼190 meV higher than those for PCBM in film and solution, respectively. This makes many of these derivatives interesting candidates as electron selective interlayers for wide-bandgap semiconductor devices, for example, for wide-bandgap perovskite solar cells, where there is currently a lack of suitable electron transport materials with shallow enough energy levels.^32−34^

#### 2.1.3 Thermal Properties

**Figure 2:**
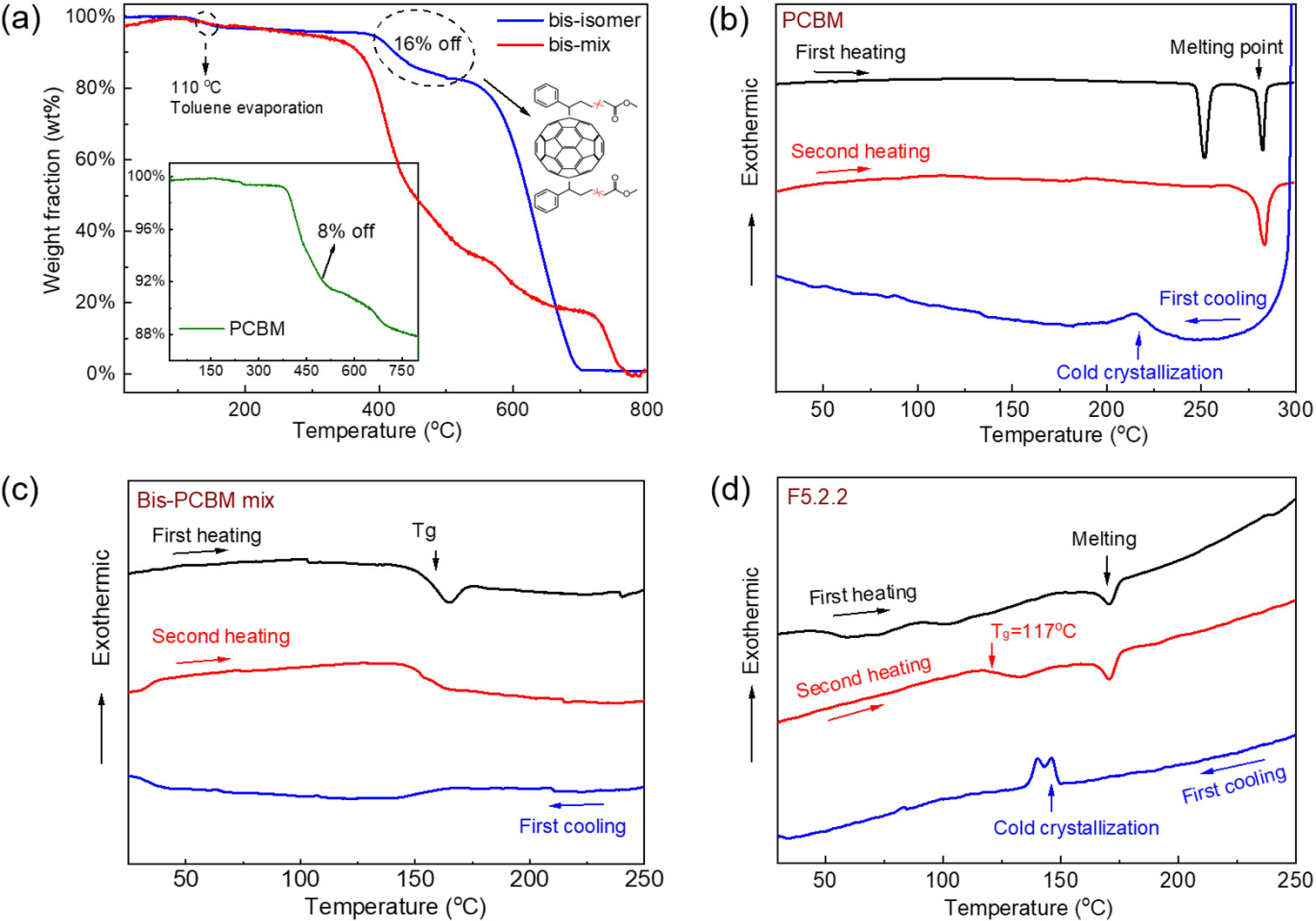
(a) Typical TGA thermograms of the bis-PCBM mixture and single isomers. The inset shows the TGA of PCBM. All measurements were carried out in a N_2_ atmosphere. (b–d) Typical DSC thermograms for (b) PCBM, (c) bis-PCBM mixture, and (d) isomer-5.2.2. The second cooling process is not shown since it is almost same as the first one. Isomers 2.1.2, 2.2, 3.2.1, 3.2.2, 3.3.2, and 5.1 show similar thermograms to the bis-adducts mixture (c). Isomers 1.1, 2.1.1, 1.2, 2.3, 6, and 7 show similar thermograms to isomer-5.2.2 (d).

Figure 2a shows TGA thermograms of PCBM and the bis-PCBM mixture compared to a typical thermogram from a single isomer, *e*-F5.1 (TGA thermograms of other isomers are shown in Figure S4A). Isolated bis-PCBM isomers exhibited notably high chemical decomposition temperatures (around 390 °C) compared to the bis-PCBM mixture (∼340 °C) and almost the same decomposition temperature as PCBM (∼395 °C). Multiple weight loss steps were observed for all samples. The first weight loss (∼1%) phenomenon for PCBM at 245 °C was studied by other researchers and attributed to the evaporation of residual solvent in the powder.^35,36^ For bis-PCBM isomer samples, the first weight loss was slightly higher (∼3%) and occurred at a lower temperature of ∼110 °C. Since this is around the boiling point of toluene, the 3% weight loss can also be assigned to toluene evaporation. The bis-PCBM mixture mass drops to 0% steadily from 340 °C, while the isolated bis-PCBM isomer exhibited only a 16% mass loss between 390 and 500 °C, before falling to zero after ∼600 °C. The mass loss of PCBM within the same range is 8%, half that of the isolated bis-PCBM isomers, suggesting that, at the same temperatures where the single side chain of PCBM is broken off, the bis-PCBM isomers simultaneously lose both side chains.^36^ To confirm that the side chains of the bis-PCBM isomers are unbroken at 110 °C, FTIR and ^1^H NMR tests were carried out for several typical isomers which had been heated to 120 °C in a N_2_-filled glovebox prior to measurement, as shown in Figure S5. These results also suggest that bis-isomers present better thermal stability than the mixture.

Two heating–cooling cycles were run for the DSC test, where the cooling process of the first and second cycles looked nearly identical. PCBM (Figure 2b) exhibits two endothermic events around 250 and 280 °C, corresponding to phase transition and melting, respectively;^36^ one broad exotherm at around 220 °C corresponding to crystallization. A broad endotherm at around 160 °C is seen in the thermogram of the bis-PCBM mixture (Figure 2c) on heating, while on cooling, a step is observed at around the same temperature. These may be associated with a glass transition temperature, suggesting that the mixture is amorphous. Indeed, no crystallization or distinct melting peaks are observed. These results are consistent with previous reports of the degree of crystallinity of PCBM and bis-PCBM mixture.^36,37^ For the isolated bis-PCBM isomers, which are expected to show better crystallinity than the mixture, two types of thermograms are observed (Figure S4B,C). Isomers 2.12, 2.2, 3.2.1, 3.2.2, 3.3.2, 5.1, and 5.2.1 show similar thermograms to the bis-adducts mixture, displaying a feature that is assigned to a glass transition at around 160 °C during the heating and cooling cycles. Above this temperature, no other thermal events are observed up to 350 °C, at which point, the samples begin to degrade slightly. These results suggest that these isomers are amorphous and we refer to them henceforth as such. The appearance of a glass transition at around 160 °C is consistent with previous reports on bis-PCBM mixture.^38^ For isomers 1.1, 1.2, 2.1.1, 2.3, 5.2.2, 5.3, 6, and 7, conspicuous phase transitions are detected in both heating and cooling cycles (Figure S4C; see Figure 2d for isomer 5.2.2). Of these, isomer 5.3 is the only bis-isomer that shows a crystallization peak during the heating process as indicated in Figure S4C. These isomers show features typical of crystalline materials, and so we refer to this set as crystalline. On first heating, an endotherm is observed at around 170 °C. On cooling, two overlapping exotherms at around 140 and 135 °C are tentatively assigned to crystallization and a second crystallization or phase transition, respectively. Interestingly, on the second heating, a step in the baseline is observed at around 117 °C. This suggests a residual amorphous component is present after cooling and that the crystallization that occurs on cooling is only partial. The temperature of this endotherm is very close to the glass transition temperature that has been observed in quenched pure PCBM.^36^ Further heating in the second heating cycle leads to an endotherm at around 170 °C, as seen on the first heating. The other isomers, namely, 3.1, 3.3.1, 3.4, and 4, show no phase transition features up to 300 °C in either heating or cooling and their crystallinity is unclear (Figure S4B). Nevertheless, those four isomers, as well as three of the crystalline isomers (1.1, 2.1.1, and 2.3), tend to aggregate in solution. We refer to this group as aggregates. The crystallization enthalpies, Δ*H*_c_, extracted from these measurements for the crystalline isomers (Figure S4D) lie between 1.3 and 2.7 J/g, much lower than the value extracted for PCBM of 10.9 J/g, indicating that they are much less crystalline than PCBM.

#### 2.1.4 Discussion of Bis-isomer Molecular Properties

As discussed above, the bis-PCBM isomers exhibit different properties: some are crystalline, some are amorphous, and some tend to aggregate in solution. Clarifying the relationship between these properties and the molecular structure is important, particularly with regard to the selection of candidates for electronic device applications. The molecular symmetry is important because it dictates the polarity of the molecule, hence influences its solubility in polar solvents, and will also influence the crystallization tendency. The point group symmetry of bis-PCBM isomers can be divided into three basic categories: *C*_1_ (7 isomers) *C*_2_ (6 isomers), and *C*_s_ (4 isomers), the exception being the two *trans*-1 isomers, which are *C*_2v_ and *C*_2h._(29) After cross-comparison of the molecular structure, point group symmetry, crystallinity, and solubility (estimated from the tendency to aggregate), a correlation may be drawn between the molecular structure and these solid-state properties. Table 1 shows that the *trans* and *e* isomers with *C*_1_ symmetry are quite amorphous, whereas those with higher symmetry crystallize or tend to aggregate. For *cis* isomers, the crystallinity does not correlate closely to point group symmetry, and most of these isomers are crystalline except for isomer 5.2.1. Overall, low symmetry bis-PCBM isomers tend to be amorphous, while symmetry appears to facilitate crystallization. However, too much symmetry can make the molecule less polar and lead to low solubility. Indeed, the two *trans*-1 isomers, with *C*_2h_ and *C*_2v_ symmetry, have the lowest solubilities of the 19 isomers. Note that although some isomers show crystalline features, the enthalpy of crystallization estimated from the DSC curves of those crystalline isomers is small, implying a relatively low degree of crystallinity relative to PCBM.

**Table 1:**
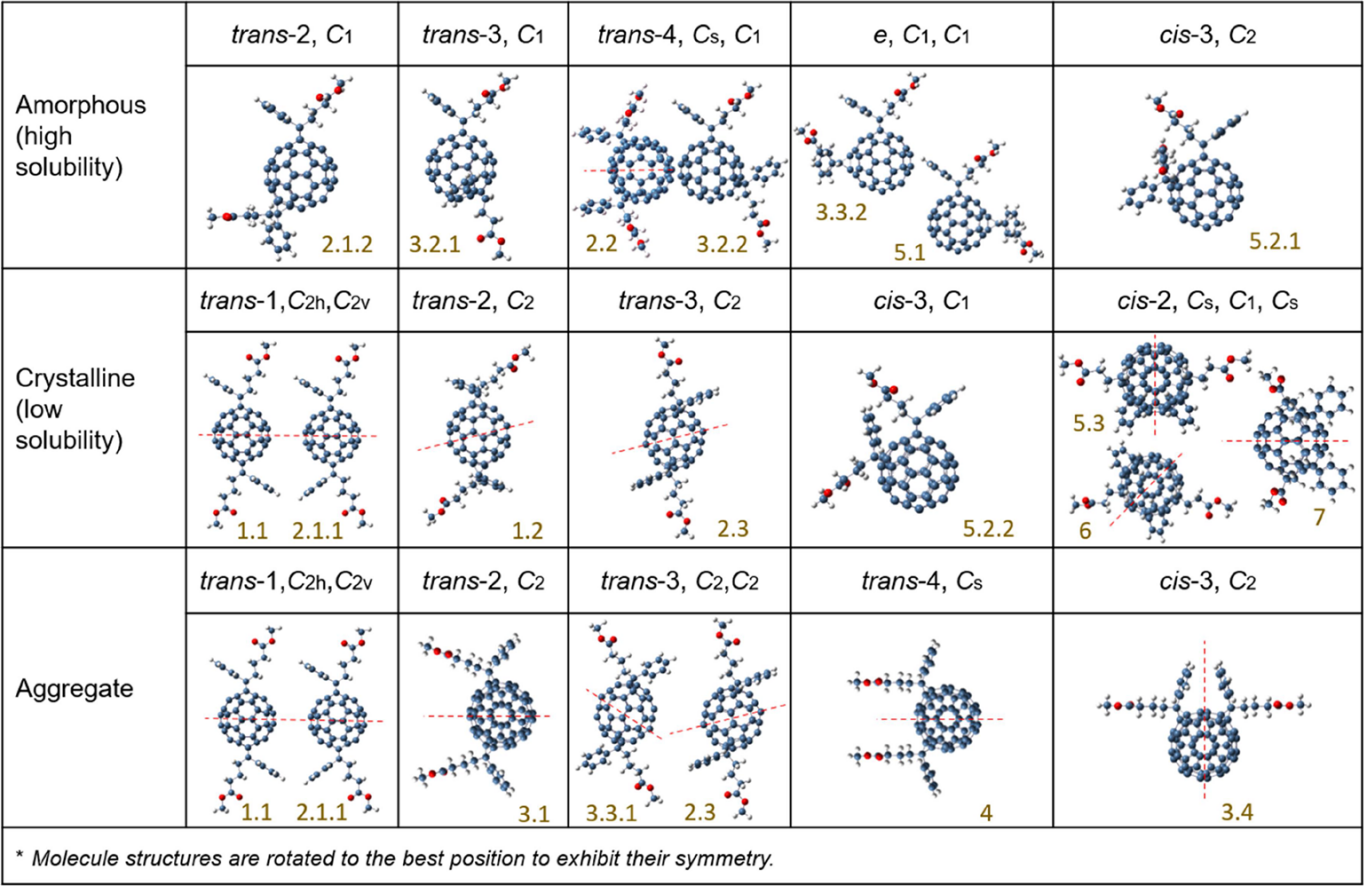
Classification of Isomers According to the Molecular Structure, Point Group Symmetry, and Crystallinity^a^

a The isomers with lower symmetry tend to be amorphous and have high solubility. Higher symmetry generally facilitates crystallization; however, too much symmetry tends to make the molecule less polar and leads to low solubility.

b Molecule structures are rotated to the best position to exhibit their symmetry.

### 2.2 Molecular Packing and Impact on the Electronic Properties

**Figure 3:**
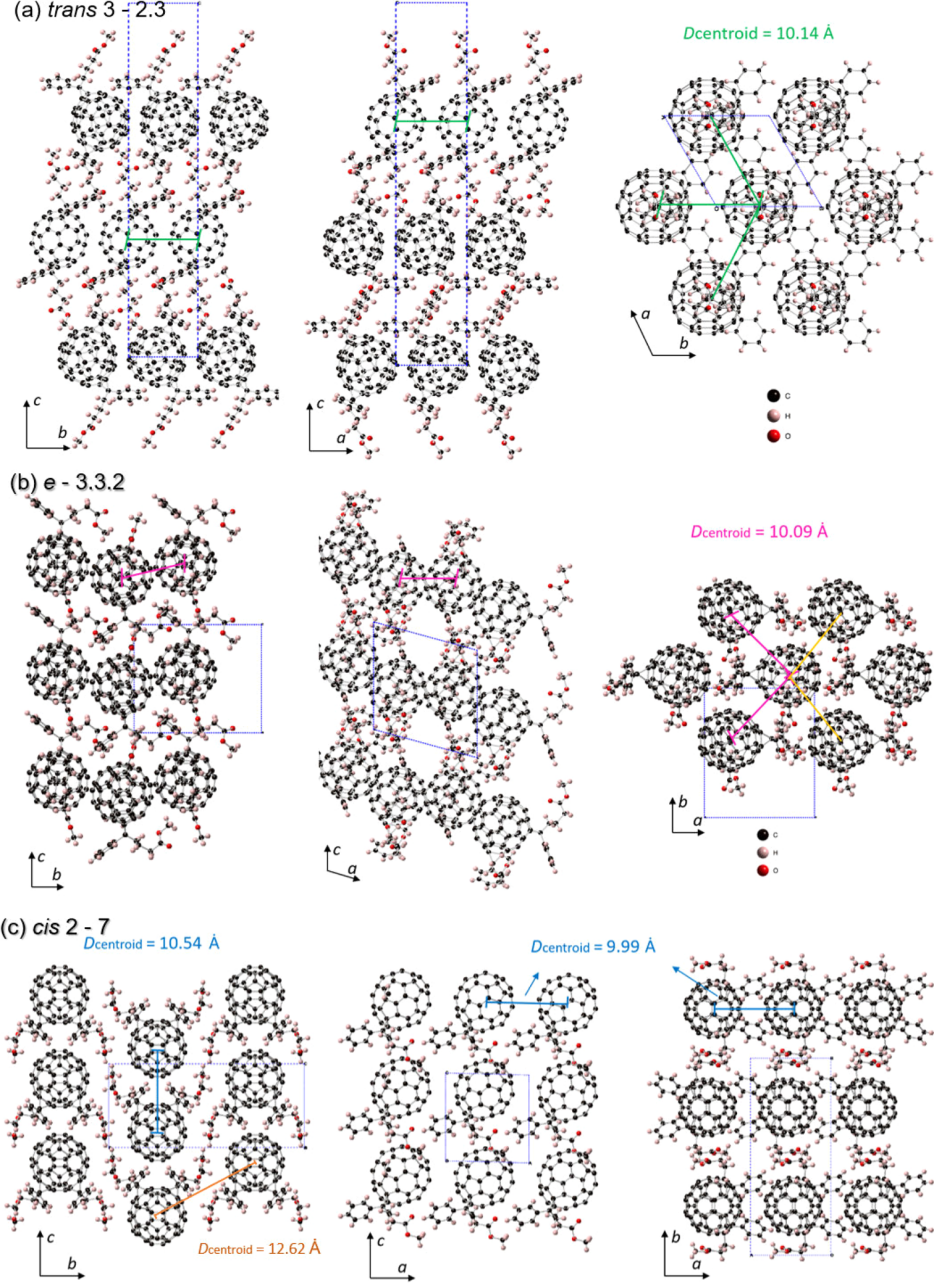
Views of the crystal structure of the isomers 2.3 (*trans-3*), 3,3,2 (*e*), and 7 (*cis-2*) from along the *a-*, *b-,* and *c*-axes. The solvent molecules are omitted for clarity. (a) Molecular packing of *trans*-3 isomer 2.3 with the trigonal unit cell in space group *P*3_1_21 containing three molecules per cell. There are six equidistant molecules around each C_60_ cage in the *a*–*b* plane with a centroid–centroid distance of 10.14 Ȧ. (b) Molecular packing of *e* isomer 3.3.2 with a monoclinic unit cell in space group P2_1_. There are two molecules per unit cell, and each molecule is surrounded by six others in the *a*–*b* plane, four of which show short centroid-centroid distances, two at 10.09 Ȧ and two at 10.23 Ȧ. (c) Molecular packing of *cis*-2 isomer 7 with a monoclinic unit cell in the P2_1_/*m* space group. There are two molecules per unit cell, and each molecule is surrounded by 8 others. The molecules are most densely packed along the *a*-axis, with centroid–centroid distances of 9.99 Ȧ.

The crystal structures of one representative isomer from each of the *trans*, *e,* and *cis*-2 groups of bis-PCBM were solved by X-ray diffraction, as shown in Figure S6. The crystals were obtained by solvent evaporation and antisolvent evaporation methods.^5,6^Figure 3 displays the molecular packing inside the crystals along the *a*-, *b*-, and *c*-axes of isomers 2.3 (*trans-3*), 3.3.2 (*e*) and 7 (*cis-2*).

As shown in Figure 3a, molecules of isomer 2.3 (*trans-3*) assemble into a trigonal unit cell in space group *P*3_1_21 containing three molecules per cell. There are six equidistant molecules around each C_60_ cage in the *a*-*b* plane with a centroid-centroid distance of 10.14 Ȧ, which is slightly longer than the value of 10 Ȧ in PCBM,^39,40^ but shorter than the value of 10.28 Ȧ in an isomer of bis-adduct C_60_(QM)_2_ that is known to be a semiconductor.^25,36^ It is therefore expected that electron transfer between two adjacent molecules in isomer 2.3 is possible. The distance between two C_60_ cages perpendicular to the *a–b* plane is approximately 16 Å, significantly longer than that in the *a-b* plane, suggesting that electron transport is essentially two-dimensional.

Figure 3b shows the molecular packing in the monoclinic unit cell of isomer 3.3.2 (*e*) with space group *P*21. There are two molecules per unit cell, and each molecule is surrounded by six others in the *a*–*b* plane, four of which show short centroid-centroid distances, two at 10.09 and two at 10.23 Ȧ. The centroid–centroid distance parallel to the *c*-axis is longer at 13.3 Å, which is expected to lead to poor carrier transport in this direction. It is noted that the single crystal of isomer 3.3.2 is tiny with poor quality, and there is no crystalline peak in the cooling process of the DSC test, suggesting that in practice this isomer is rather amorphous.

Figure 3c shows the molecular packing in the monoclinic unit cell of isomer 7 (*cis-2*). The unit cell shows *P*2_1_/*m* space group symmetry and contains two molecules per unit cell. Each bis-adduct molecule is surrounded by eight others. The molecules are most densely packed along the *a*-axis, with a centroid–centroid distance of 9.99 Ȧ. The centroid–centroid distances along the *c* and *b*-axes are longer at 10.54 and 12.62 respectively, suggesting that electron transport may have a single preferred direction.

The solved crystal structures of the bis-PCBM isomers indicate that the electron transport within the fullerene cage assembly is likely to show low dimensionality, with the 2.3 (*trans-*3) and 3.3.2 (*e*) isomers likely to show two-dimensional transport, while the highly symmetric 7 (*cis*) isomer is likely to show one-dimensional transport along the *a* direction. Two- or lower dimensional transport is common in organic semiconductors;^41^ however, three-dimensional transport is often observed in PCBM crystals.^41,42^

To confirm the dimensionality of charge transport in the solved crystal structures, we carried out charge transport simulations, the results of which are shown in Table 2. A hopping mechanism was assumed for the transfer of electrons between fullerene cages, allowing hopping rates to be computed using semiclassical Marcus theory. Values for the electronic structure parameters in the Marcus nonadiabatic charge transfer rate equation were based on those adopted in ref (14). along with a total reorganization energy of 0.2 eV (results based upon an estimated total reorganization energy of 0.5 eV are shown in the ESI and predict similar trends). Mobilities were found by solving the steady state master equation for each of the crystal structures (see Supporting Information for details). The results show that electron transport is significantly anisotropic in all cases. Crystals 2.3 (*trans-3*) and 3.3.2 (*e*) exhibit two-dimensional electron transport, with the *a* × *b* direction being approximately nontransporting in both cases. Electron transport in crystal 7 (*cis-2*) is closer to being one-dimensional, as expected, with *b* × *c* being the fastest direction, *a* × *b* being eight times slower, and *c* × *a* being several orders of magnitude slower still.

**Table 2:** Simulated Electron Transport Mobilities for Three Different Crystal Structures at Different Field Directions, Quoted in cm^2^ V^–1^ s^–1^, Assuming a Total Reorganization Energy of 0.2 eV

**field direction**	**crystal 2.3 (***trans-3***)**	**crystal 3.3.2 (***e***)**	**crystal 7 (***cis-2***)**
*b* × *c*	2.85	0.82	2.06
*c* × *a*	2.85	1.24	3 × 10^–5^
*a* × *b*	1 × 10^–9^	6 × 10^–6^	0.25

### 2.3 Field-Effect Transistor and Photovoltaic Device Performance

We proceed to explore the correlation between molecular properties and the (opto)electronic properties of thin films of the different isomers, first considering electron transport in organic field-effect transistors (OFETs) and then the electrical response of polymer:fullerene solar cell devices. Since mixtures of bis isomers suffer from energetic disorder leading to poor electron mobility,^43^ we may expect isolated bis-isomers to result in better OFET mobilities and higher fill factors in solar cells^9^ than the isomer mix.

#### 2.3.1 OFET

**Figure 4:**
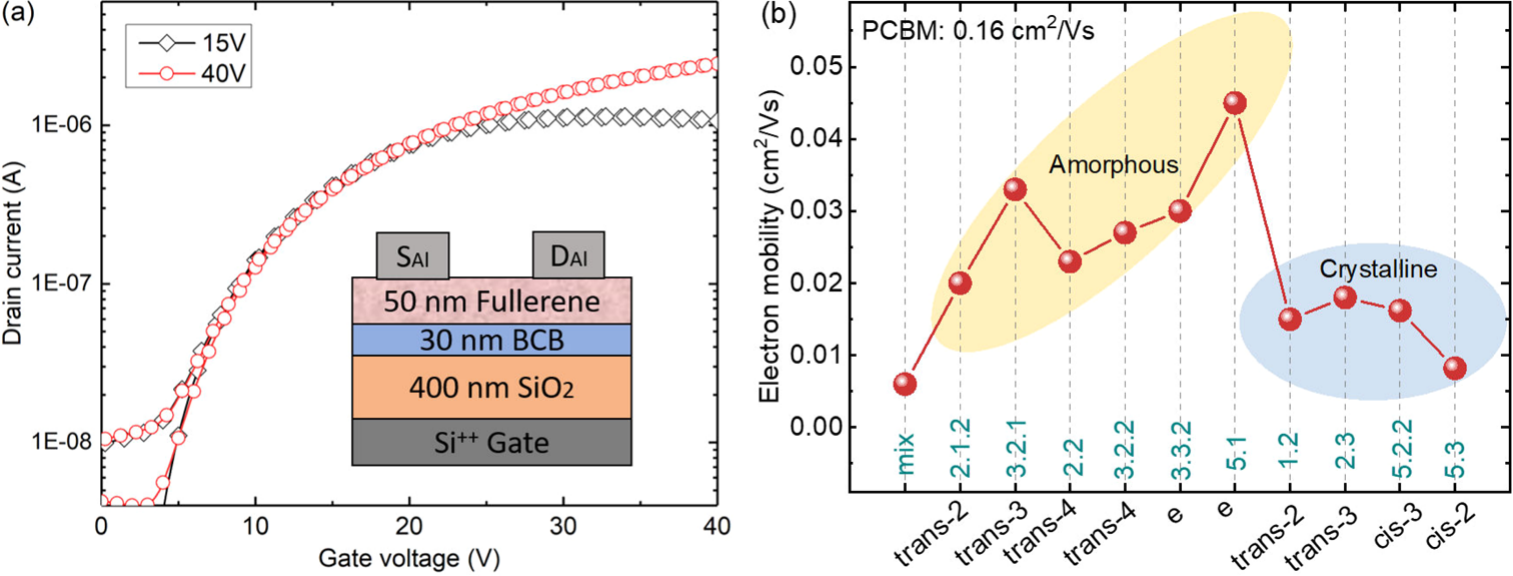
(a) Transfer characteristics of a typical fullerene transistor (F3.2.1) with a channel length of 30 μm and a width of 1000 μm. The inset shows the bottom-gate, top-contact transistor architecture. (b) Calculated electron mobilities for different isomers in the saturation regime. The isomer 7 is not shown since it did not form a working OFET device, leading to very low mobility less than 8 × 10^–4^ cm^2^/(V s).

The influence of side chain position on crystallinity and the packing of fullerene cages are expected to be reflected in the electron mobility. To explore this, we fabricated OFET devices employing the bottom-gate, top-contact transistor architecture (inset of Figure 4a). Figure 4a shows the evolution of drain current versus gate bias, measured at drain voltages of 15 and 40 V. The measurement at 15 V corresponds to the linear operating regime, while the curve measured at 40 V relates to the saturation regime. Figure 4b shows the maximum resulting electron saturation mobility for bis-PCBM isomers, which varies from 6 × 10^–3^ cm^2^/(V s) for the bis mixture to 4.5 × 10^–2^ cm^2^/(V s) for the *e* isomer 5.1. All single isomers outperform the bis-PCBM mixture, potentially making them excellent candidates as electron transport layers in a range of perovskite solar cells, where the energy levels of the bis-PCBM can be tuned to that of the perovskite layer to reduce interfacial losses.^18^ We note that the highest electron mobility of any single bis-PCBM isomer is still ∼3 times lower than that of PCBM, which is 1.6 × 10^–1^ cm^2^/(V s) using the same method (the measured electron mobility of PCBM is similar to literature values^44^).

Interestingly, the amorphous bis-isomers, for which the second side chain tends to lie near the equator area, all show higher electron mobilities than the more crystalline ones. While crystallization is often associated with good transport, this behavior could be explained by the relatively low degree of crystallization (indicated by the enthalpy of crystallization) of pure bis-isomers, which may be inadequate to build good transport networks. In addition, the dimensionality of packing seems to have a strong influence on the charge transport. For example, simulations of charge transport on the single crystal structure of the *cis*-isomer 7 showed lower dimensionality than the *trans* and *e* isomers considered. This is roughly consistent with the behavior shown by the OFET mobilities in Figure 4b. These results suggest that both crystallinity and packing dimensionality play important roles in determining charge transport properties.

#### 2.3.2 Organic Solar Cell Devices

We now assess the performance of OPV devices made by using each of the bis-PCBM isomers as the acceptor. With reduced energetic disorder relative to mixtures of isomers, single bis-PCBM isomers can be expected to yield higher fill factor (FF) and open-circuit voltage (*V*_oc_) when used as acceptors in bulk heterojunction (BHJ) solar cells. An interesting question is whether the inability of the isomer mixture to crystallize could explain the poor performance of solar cells made using bis-PCBM isomer mixes in some previous studies.^11^ The availability of distinct isomers to test as electron acceptors also allows us to explore the correlation between the molecular properties and OPV performance by using those isomers.

**Figure 5:**
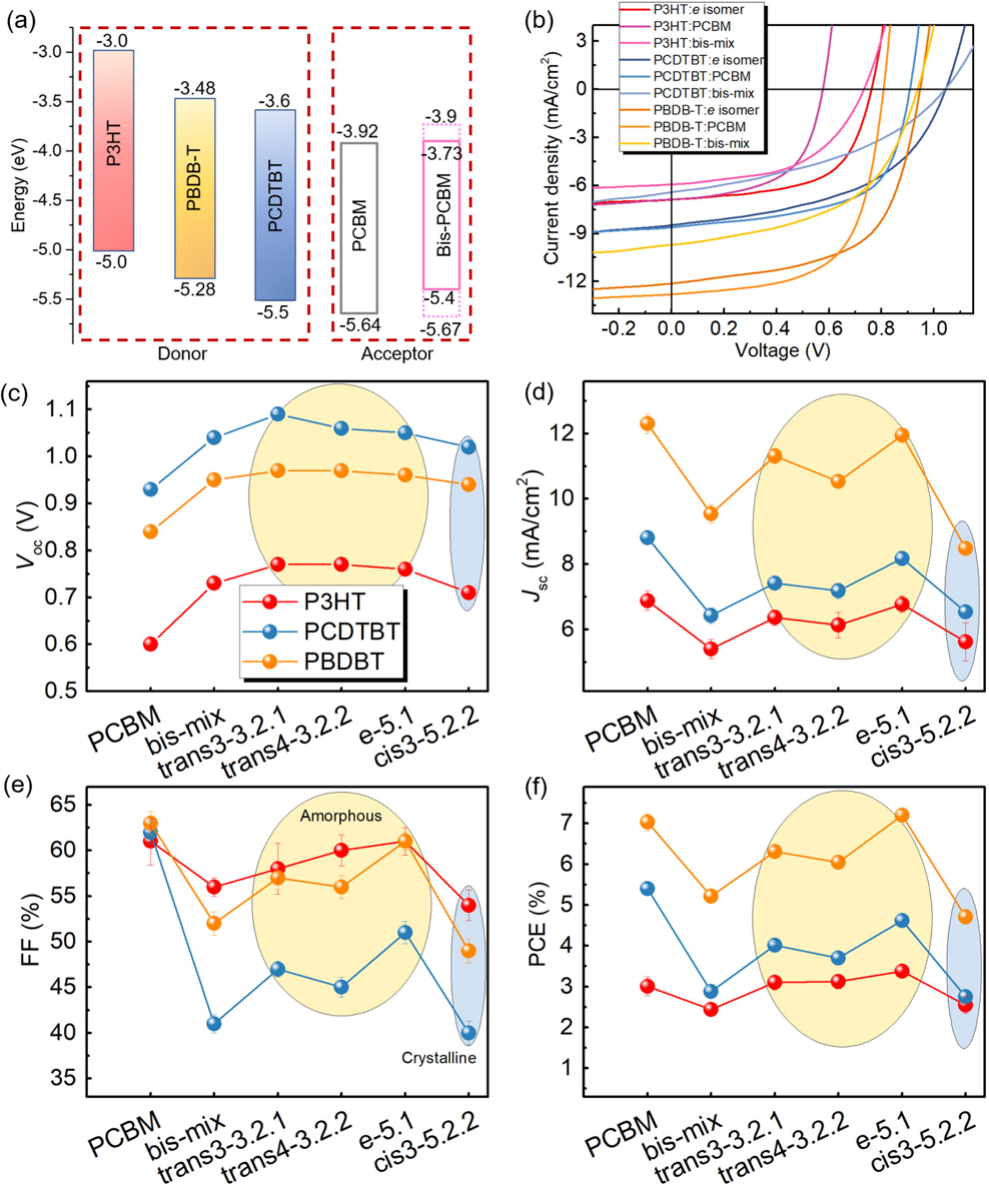
(a) Energy levels of the polymer donors and fullerene acceptors PCBM and bis-PCBM isomer.^46,47^ (b) *J*–*V* curves of polymer:fullerene devices based on PCBM, bis-PCBM mixture, and selected isomers. (c–f) Extracted and averaged device parameters using the arithmetic average of each quantity among the sample including PCE, *J*_sc_, *V*_oc_, and FF of the different polymer:fullerene devices. The isomers 3.2.1 (*trans*-3), 3.2.2 (*trans*-4), and 5.1 (*e*) are amorphous, while the isomer 5.2.2 (*cis-*3) is crystalline.

Three polymers with different crystallinity, crystalline poly(3-hexylthiophene-2,5-diyl) (P3HT), amorphous poly[N-9′-heptadecanyl-2,7-carbazole-*alt*-5,5-(4′,7′-di-2-thienyl-2′,1′,3′-benzothiadiazole)] (PCDTBT), and semicrystalline poly[(2,6-(4,8-bis(5-(2-ethylhexyl)thiophen-2-yl)-benzo[1,2-b:4,5-b’]dithiophene))-*alt*-(5,5-(1′,3′-di-2-thienyl-5′,7′-bis(2-ethylhexyl)benzo[1′,2′-c:4′,5′-c’]dithiophene-4,8-dione)] (PBDB-T), were blended with the bis-PCBM isomers separately to fabricate solar cells with the layer structure ITO/PEDOT:PSS/polymer:fullerene/interlayer/Al (Figure 5a), where the electron selective interlayer was Ca or PFN depending on the polymer. All bis-PCBM isomers were blended with P3HT under the same conditions except for those that easily aggregate and do not form a good solution with the polymer. Selected isomers from each of the *trans*, *e,* and *cis* groups, as well as the bis-PCBM mixture and PCBM, were blended with PCDTBT and PBDB-T. The device performance data are summarized in Tables S3 and S4. Details of the device fabrication, including blend ratios and processing methods, can be found in the Methods Section. We note here that all P3HT:fullerene blend weight ratios are chosen to be 1:1 according to the range of those used previously,^45^ although this ratio might not be the optimum for all isomers. For example, 1:1.2 was previously used for the P3HT:bis-PCBM mixture.^1^Figure 5b shows typical *J*–*V* curves for the different polymer:fullerene devices, demonstrating the variation between the blends. As expected, replacing PCBM with the bis-PCBM mixture raises *V*_oc_ but degrades the FF and short circuit current density (*J*_sc_). Interestingly, using a single isomer rather than the mixture increases both the *J*_sc_ and FF to approach the PCBM device values. For better comparison, device parameters averaged over at least eight nominally identical devices are extracted from the *J*–*V* curves, as shown in Figure 5c–f.

**Figure 6:**
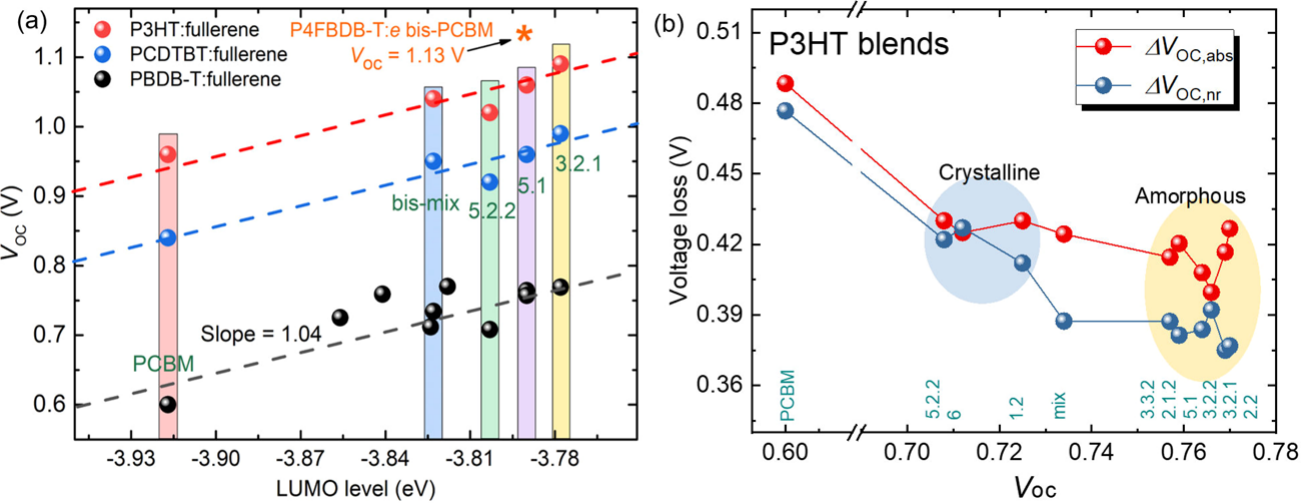
Voltage loss analysis. (a) Open circuit voltage as a function of the LUMO level of fullerene; (b) voltage losses as a function of open circuit voltage. The *V*_oc_ value 1.13 V of P4FBDB-T:bis-PCBM blend is from ref (48).

We begin by discussing the results for the P3HT:fullerene blends. Here, all single isomer-based devices show superior PCE to that of the bis-PCBM mixture, and most of the devices using isomers show slightly higher PCE than the PCBM-based device. The *e* isomer 5.1 leads to the highest PCE of 3.38% (see Table S3). Since the *J*_sc_ and FF of single-isomer devices are comparable to or slightly lower than those of the PCBM device, we can infer that it is the improved *V*_oc_ that leads to the superior PCE, which can be understood to result from the high-lying LUMO of the bis-PCBM isomers (−3.75 to −3.86 eV by CV) relative to PCBM (−3.92 eV). Figure 6a shows the correlation between *V*_oc_ and LUMO energy of the acceptor, where for each polymer the data follow a simple relationship whereby *V*_oc_ increases with the acceptor LUMO and hence with the donor HOMO-acceptor LUMO energy gap. Among the P3HT:bis-PCBM isomer devices, those based on amorphous isomers exhibit a higher PCE and higher values of most photovoltaic parameters than those based on crystalline isomers (Table S3). It is not intuitively obvious why this should be, but we notice that the *V*_oc_ of the device made from crystalline 5.2.2 lies below the value expected if *V*_oc_ depended only on the fullerene LUMO energy (Figure 6a) and that the FF is significantly lower, and the *J*_sc_ slightly lower, than for devices made from amorphous polymers. In our study, the P3HT:bis-PCBM mixture shows a lower PCE than the P3HT:PCBM device in contrast to ref (1). We assign this difference to the lower fullerene mass ratio used in our work, which we kept constant at 1:1 for a fair comparison.

To further investigate the variation in *V*_oc_ with isomer, we carried out *V*_oc_ loss analysis of the selected P3HT devices by analyzing electroluminescence (EL) and sub-band gap external quantum efficiency (EQE) measurements on the blend devices.^48,49^ The data are shown in Figure S7, and the methods are described in the Supporting Information section. The results in Figure 6b demonstrate, first, that all devices made with bis-isomers show lower nonradiative (Δ*V*_oc,nr_) and absorption broadening (Δ*V*_oc,abs_) voltage losses than the PCBM device and second, that the devices based on crystalline isomers show slightly higher Δ*V*_oc,nr_ losses than those based on amorphous isomers. The reduced Δ*V*_oc,abs_ for bis-PCBM isomers relative to PCBM is expected from the reduced energy difference between the heterojunction charge transfer (CT) state energy and the optical band gap (here due to the polymer) as the offset between donor and acceptor LUMO energies decreases. The trend in Δ*V*_oc,nr_ is consistent with the energy gap law, which predicts that materials with lower CT state energy (compare the EL peak energy in Figure S7b with the Δ*V*_oc,nr_ in Figure 6b) suffer more nonradiative recombination.^50^ We also extracted the Gaussian width and peak energy from the EL spectra of the devices, as shown in Figure S8. The Gaussian width of the EL provides information about the amount of disorder in the devices; we find that isomer 5.2.2 and the bis-PCBM mixture lead to the highest Gaussian width, indicating larger energetic disorder relative to that of other bis-isomers. Moreover, the more crystalline bis-isomers show the EL peak position at a lower energy, indicating a lower energy of charge transfer states. Among crystalline bis-isomers, 5.2.2 also shows the lowest peak energy and widest Gaussian width (hence the largest energetic disorder). Larger disorder together with a lower energy of CT states can lead to extra voltage losses,^50,51^ which may account for the additional voltage loss of ∼50 meV in the case of 5.2.2 compared to 5.1 (the best performing bis-isomer).

To further understand the device performance differences among different bis isomers, scanning electron microscopy (SEM) imaging was first carried out to study the device morphology. The images show that, when each is blended with P3HT, the amorphous bis-PCBM isomers form a similar morphology to PCBM, while films with bis-PCBM mixture or the crystalline isomer 5.2.2 show a coarser surface microstructure (Figure S9A).

The lower *J*_sc_ and FF of the crystalline isomer-based device are consistent with the lower OFET mobility (Figure 4b) and can be assigned partly to increased energetic disorder and potentially to a lower-dimensional electron pathway inside the fullerene phase relative to that of amorphous isomers (Figure 3). The analysis here could explain the relatively low *V*_oc_ and FF of devices based on the bis-PCBM mixture and 5.2.2. The results mentioned above confirm our expectations, based on previous studies, that for the crystalline, deep LUMO polymer P3HT, bis-PCBM devices can outperform devices based on PCBM. The effect of bis-PCBM isomers on devices made from other less crystalline polymers is less obvious. Next, we replace crystalline P3HT with either amorphous PCDTBT or semicrystalline PBDB-T, to further understand how the crystallinity of the bis-isomer affects device performance. Several earlier observations on polymer:fullerene systems suggested that good performance was associated with the ability of at least one component to form a robust charge transport network through its ability to crystallize.^52,53^ For example, poor performance of devices made from an amorphous polymer, PCDTBT, blended with a fullerene bis-adduct mixture, was assigned to poor electronic properties due to the inability of either component to crystallize.^11^ On this basis, we may expect isolated bis-isomers to outperform the bis-PCBM mixture when blended with PCDTBT and, moreover, that more crystalline bis-isomers should lead to better device performance than amorphous ones. It is also interesting to observe whether a greater polymer crystallinity benefits the device fill factor. This set of devices thus allows the effects of both the polymer and fullerene crystallinity to be investigated.

First, for both PCDTBT and PBDB-T, we observe an increase in *V*_oc_ correlated with the isomer LUMO energy, exactly as for P3HT (Figure 6a). We also observe that the trend in photovoltaic parameters (*J*_sc_, *V*_oc_, FF, and PCE) for the device based on PCDTBT or PBDB-T show similar trends as for P3HT (Figure 5c–f, data see Table S4). While the individual isomers tend to outperform the bis-PCBM mixture, devices made with the crystalline isomers show worse performance than those made with amorphous isomers but similar to the trend observed for P3HT blends. Isomer 5.1 shows the highest PCE, while crystalline isomer 5.2.2 performs the worst. This suggests that while separating the isomers does benefit performance, possibly through reduced energetic disorder, using more highly crystalline bis-isomers does not improve device performance. However, the relatively limited degree of crystallinity of the bis-PCBM isomers (Figure S4) probably compromises their performance. Regarding the effect of polymer crystallinity, we observed a positive effect of polymer crystallinity on the device, whereby PBDB-T-based devices with any bis-isomer showed the highest FF. It is also noted that the AFM images of the PCDTBT devices (see Figure S9B) exhibited quite amorphous morphology.^23^ The blends with crystalline fullerene (PCBM and isomer 5.2.2) and bis-mix show slightly higher roughness (∼16 nm) than that of the blends with amorphous isomers (<10 nm), which is similar to the situation of the P3HT blends, where the bis-mix and isomer 5.2.2 show a coarser surface microstructure. Finally, we observe that, while no PCDTBT:bis-adduct blend outperformed the PCDTBT:PCBM reference, in the case of the PBDB-T devices, the device made with isomer 5.1 outperformed the PBDB-T:PCBM reference with a PCE of 7.2% compared to 7.0%. To the best of our knowledge, this is the best performing organic solar cell with a bis-adduct acceptor to date.^54^ The highest *V*_oc_ of around 1.1 V was achieved in our study using a blend of PCDTBT and 3.2.1 (*trans-*3), while the *V*_oc_ can be further increased via optimizing the polymer, for example, a high *V*_oc_ of 1.13 V was demonstrated for a P4FBDB-T:bis-PCBM blend.^48^

The observation that all polymers, regardless of the degree of crystallinity, favor less crystalline bis-PCBM isomers is surprising. This behavior may be explained by the low degree of crystallinity shown by the bis isomers compared to PCBM, whereby the formation of small crystals may result in isolated charge traps and hence increased energetic disorder, rather than robust transport pathways. An additional factor may be the low-dimensional packing in some crystalline isomers, as seen in isomer 7, and the resulting effect on transport.

## 3 Discussion

Isolated bis-PCBM isomers with single side chain configurations are regarded as better candidates for high performance polymer:fullerene blend devices thanks both to their higher lying LUMO (and hence larger *V*_oc_) than PCBM devices and to their lower energetic disorder compared to isomer mixes. Both features are evidenced in the devices studied here [narrow EL peak widths (Figure S8a) confirm low disorder, while the higher *V*_oc_ values for bis-isomers than PCBM (Figures 5c and 6a) reflect their higher lying LUMO].

**Figure 7:**
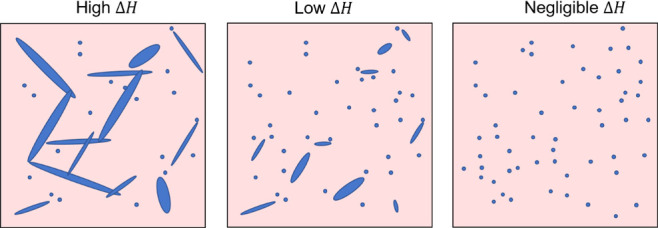
Schematic of the proposed fullerene structure formation in polymer films. High enthalpy of crystallization encourages large crystals to form (left); low Δ*H* of weakly crystalline fullerene limits the crystal size and only able to form small crystals (middle); and amorphous fullerene has negligible Δ*H* and cannot form crystallites (right).

The best charge transport behavior and PV performance are achieved by using amorphous rather than crystalline isomers, in contrast to the general understanding that the crystallinity of the fullerene component assists in the formation of a robust transport network and facilitates charge transport.^52,53^ This may be related to the fact that the crystalline bis-isomers display a much lower crystallization enthalpy (2.0 J/g) compared to that of PCBM (10.9 J/g) under similar conditions. This may promote the formation of multicrystalline phases or small crystallites, either of which tends to show higher energetic disorder (and more trapping) due to the variation in electronic coupling between molecules. Figure 7 illustrates a hypothetical trend in microstructure that is consistent with the observations: in the case of PCBM (left), the high enthalpy of crystallization encourages large crystals to form, sufficient to form a network for electron transport through the polymer. Weakly crystalline bis isomers are able to form small crystals, but low Δ*H* limits their size with the result that they form isolated electron traps inhibiting transport. In the case of amorphous bis isomers, (right) fullerene crystallites do not form, avoiding such electron traps, and charges move by hopping between isolated fullerenes dispersed in the polymer. An additional effect may be the less isotropic molecular packing of the bis-isomer crystals compared to PCBM: those crystals that were formed supported close packing in only one or two dimensions, rather than 3D as for PCBM.^42^

As shown in our transport simulation results, more anisotropic crystalline packing leads to lower dimensional transport. Such anisotropic packing could explain the poor electron transport in crystalline bis-PCBM isomers and help to explain why the photocurrent and FF are poor in OPV devices made from crystalline isomers, regardless of the choice of polymers.

We observe that the isomer crystallinity shows certain correlations with the symmetry of the molecular structure. Molecules with no symmetry tend to be amorphous, while moderate symmetry can help to improve crystallinity. The high-symmetry materials (some of the *C*_2_ and *C*_s_ isomers) have low solubility and tend to precipitate into powder in solution. Those crystalline isomers that show tolerable solubility are the *trans*-2 isomers and *cis* isomers, whose side chains are near the fullerene pole positions. In the bis-PCBM isomers studied, molecules with higher symmetry generally show higher crystallinity but worse mobility and solar cell efficiency. Interestingly, we find that this observation seems to be general in the literature where we note similar results based on other higher-adduct fullerenes.^15,25^ As listed in Table 3, several isomers with different point group symmetry from three types of bis-adducts fullerene were selected to summarize their symmetry, electron mobility, and device PCE. In the isomers in the three groups of C_60_(QM)_2_, [60]C6BA, and [60]C4BA, those with high symmetry exhibit lower electron mobility and device PCE compared to their low symmetry counterparts. Therefore, the choice of point group symmetry could be one important criterion for future higher adduct fullerene synthesis and application.

**Table 3:**
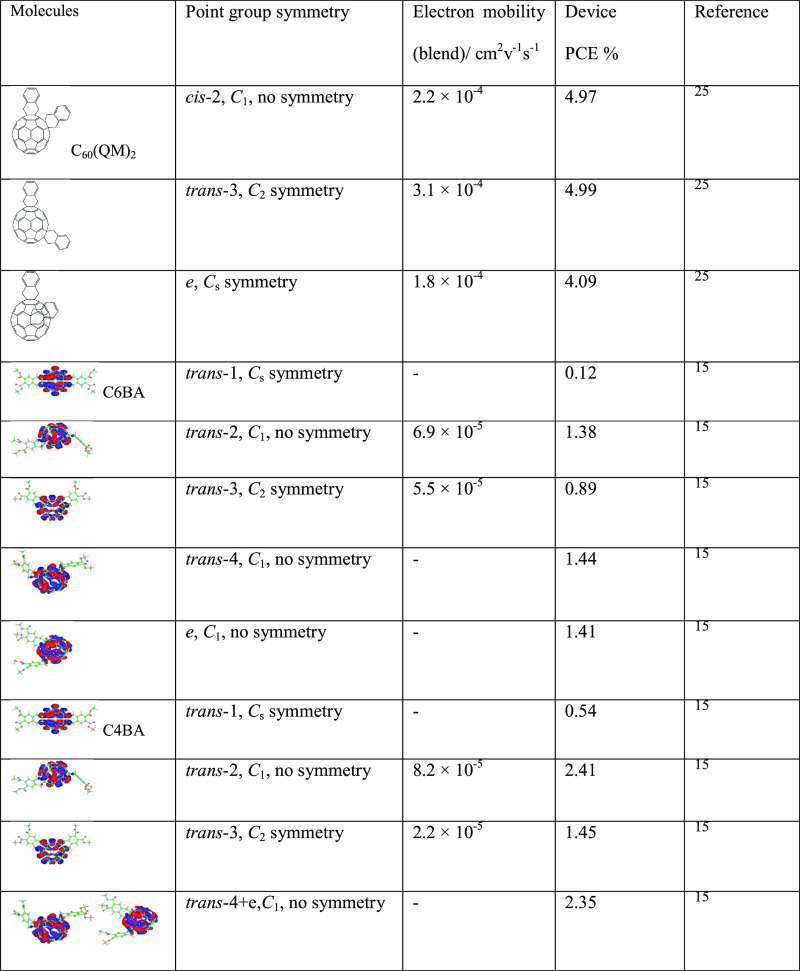
Comparison of the Molecular Syncroscopic and Device Performance (After Blending with P3HT)^a^

a Three bis-adducts fullerenes, C_60_(QM)_2_,^25^ [60]C6BA, and [60]C4BA are summarized.^15^

## 4 Conclusions

In this work, we demonstrated the purification of isometric mixtures of bis-PCBM into 19 regio- and/or stereoisomers and characterized the different isomers in terms of energy levels, symmetry, crystallinity, and molecular packing. We find that the LUMO of the bis-isomers can be tuned to be ∼170 and ∼100 meV shallower than that of PCBM and the bis-mixture, respectively. Importantly, the electron mobilities of all bis-isomers, as measured using OFET devices, are higher than those of the bis-mixture. We find that amorphous isomers generally show a higher mobility than crystalline isomers, with the best performing showing an electron mobility of 4.5 × 10^–2^ cm^2^/(V s), almost an order of magnitude higher than the bis-PCBM mixture. We propose that the shallow LUMO, in addition to the high mobility, makes these bis-isomers excellent candidates as electron transport layers for reducing interfacial losses in perovskite solar cells and, in particular, for wide-bandgap perovskite solar cells where there is currently a lack of materials with suitably shallow energy levels.

We further investigated the impact of bis-isomer molecular properties on BHJ solar cell devices with three different polymer donors, P3HT, PCDTBT, and PBDB-T. Like in OFETs, we find that more amorphous bis-isomers perform better in solar cell devices than crystalline ones, with the best performing showing a PCE of 7.2% (with PBDB-T as donor), higher than the corresponding PCBM reference, and the best performing solar cell with a bis-PCBM acceptor to date, to our knowledge. Finally, we propose that the solid-state properties of bis-PCBM isomers may be related to their molecular symmetry, with a lower symmetry leading to more amorphous bis-isomers, less energetic disorder, and higher dimensional electron transport, resulting in higher electron mobility and OPV device performance relative to more symmetric isomers.

## 5 Methods

### 5.1 Theoretical Calculations

Geometry optimization and electronic structure calculations for the bis-PCBM isomers and PCBM were performed using density functional theory at the B3LYP/6-311G (2df, 2pd) level. The calculations were carried out with Gaussian 09 software package under vacuum.^9^

### 5.2 UV–vis and UPS

The UV–vis spectra of the bis-PCBM isomers were recorded on a Shimadzu UV-2600 spectrophotometer between wavelengths of 325–800 nm. The scanning rate, step size, and path length were 30 nm/min, 0.2 nm, and 10 mm, respectively. The UPS measurements were carried out by an Axis Supra instrument (Kratos Analytical, UK) with a monochromatic Al Kα X-ray source and a He discharge lamp (HeI, 21.22 eV). The films for UV–vis test were spin-coated on quartz, and the ones for UPS test were spin-coated on ITO substrates (∼ 50 nm).

### 5.3 TGA/DSC

TGA measurements were conducted on a Thermogravimetric balance from TA Instruments (TGA-Q500). Around 5 mg of sample was weighed into a platinum pan. The samples were heated in nitrogen at a rate of 10 °C/min from 25 to 800 °C and the sample kept at 800 °C for 10 min to ensure complete decomposition. DSC measurements were carried out with a TA Instruments calorimeter (mode DSC2A-00503). Approximately 5 mg of sample was weighed into an aluminum pan, and the pan was sealed by pressing. The samples were left to equilibrate at 25 °C for 20 min, then heated under nitrogen at a rate of 10 °C/min to 300 °C, held at this temperature for 5 min, and then cooled at a rate of −10 °C/min to 25 °C. Two heating–cooling cycles were performed for each sample.

### 5.4 Transistor Fabrication

OFETs were constructed on highly doped silicon substrates (2 × 2 cm) covered with 400 nm SiO_2_, which were precleaned by sonication in acetone, DI water and IPA for 10 min each prior to use and treatment by ozone under UV for 15 min. A divinyltetramethyldisiloxane-bis(benzocyclobutene) (BCB) layer with a thickness of 30 nm was spin coated on top of the substrate in a nitrogen filled glovebox. The BCB layer was then annealed from 100 to 280 °C gradually and kept at 280 °C for 60 min.^44^ When the BCB layer had cooled to 100 °C, the bis-PCBM isomer solution (20 mg/mL in CB) was spin coated onto the surface to a thickness of around 50 nm. To complete the device, a 50 nm thick layer of Al was evaporated onto the fullerene layer under a vacuum of 10^–6^ Torr through a shadow mask to serve as source and drain electrodes. The shadow mask defined the transistor channel length (*L*), varying from 30 to 100 μm and width (*W*) of 1000 μm. Current–voltage characteristics of the transistors were measured at room temperature in a nitrogen-filled glovebox using an Agilent B2902A semiconductor parameter analyzer.

### 5.5 Solar Cell Fabrication

The solar cell devices were fabricated with the normal architecture of an ITO/PDEDOT:PSS/active layer/interlayer/top electrode. All layers from solution were fabricated by spin coating. Metal interlayers and top-electrodes were thermally evaporated. The ITO substrates (1.2 × 1.2 cm) were cleaned by sequential sonication in acetone, diluted detergent water, DI water and IPA for 15 min at each cleaning step. Then the PEDOT:PSS layer was spin-coated and annealed at 150 °C for 20 min. After this, the prepared PEDOT:PSS coated ITO substrates were transferred into a N_2_ filled glovebox (<0.1 p.p.m O_2_ and H_2_O) for active layer coating. The bis-PCBM isomers were purified using HPLC several days before device fabrication. Since the bis-PCBM isomers were easier to oxidize under air compared to PCBM, each of the isomer solutions from the HPLC was first concentrated in a rotary evaporator to a hypersaturated state and then placed in a vacuum drying oven to remove other solvents.

For the P3HT:fullerene devices, P3HT and PCBM/bis-PCBM isomers were weighed in a ratio of 1:1 to a vial with CB inside to form a total concentration of 18 mg/mL. After overnight heating at 80 °C and cooling to room temperature, the active layer was deposited and annealed at 110 °C for 10 min. Transferred the films to the evaporator inside the same glovebox and deposited Ca (20 nm) and Al (80 nm) at a vacuum level of 1 × 10^–6^ Torr.

For the PCDTBT:fullerene devices, the solid PCDTBT and bis-PCBM isomers were dissolved in CB with a weight ratio of 1:2 forming a solution with concentration of 20 mg/mL. After overnight heating at 85 °C and cooling to room temperature, the active layer was spin coated to form a layer with thickness of 80–90 nm. The films were carried out THF SVA treatment: a glass Petri dish containing ∼1.5 mL of THF was prepared with a clean glass slide placed at the bottom; the active layer film was placed onto the slide in the Petri dish and covered the dish with a lid quickly; and the film was taken out after 90 s. A PFN thin layer with a thickness of 5 nm was further spun on the active layer. Then transferred the films to evaporator for Al (100 nm) deposition under 1 × 10^–6^ Torr.

For the PBDB-T:fullerene devices, the donor: acceptor weight ratio was set as 1:1. The solid materials were dissolved in a solution mix of 97 vol % CB and 3 vol % 1,8-diiodooctane (DIO) to form a total concentration of 20 mg/mL. To completely dissolve the polymer, the solutions were heated on a hot plate at 80 °C for at least 12 h. When the solutions cooled to room temperature, active layers were spin-coated on the prepared ITO/PEDOT:PSS substrates. When the active layer surface was dry, pure methanol was then spun onto the films at a seed rate of 4000 rpm for 40 s to remove residual DIO. After that, a thin PFN layer (5 nm) was further spin-coated on the films to serve as the interlayer. Finally, the devices were transferred to the evaporator for top electrode Al deposition (100 nm).

The size of each obtained solar cell was 0.045 cm^2^. Current density–voltage (*J*–*V*) characteristics were measured by a Keithley 2400 Source measure unit and a xenon lamp with AM 1.5G filters and 100 mW/cm^2^ illumination solar simulator (Oriel Sol3A Class AAA). The illumination intensity was adjusted with a calibrated KG-5 silicon diode from Newport.

## Supporting Information Available

Structure and property of bis-PCBM isomers, single crystal X-ray diffraction, solar cell performance, and charge transport simulations (PDF)
