# Supplementary material for: Structure–Property Relationships for the Electronic Applications of Bis-Adduct Isomers of Phenyl-C61 Butyric Acid Methyl Ester
Source: Chem Mater. 2023 Dec 28;36(1):425–38. doi: 10.1021/acs.chemmater.3c02353 (PMC10782444; doi:10.1021/acs.chemmater.3c02353)
Supplement: Supplementary file 1 — cm3c02353_si_001.pdf [file cm3c02353_si_001.pdf]

Supplementary Information of

Structure-property relationships for the electronic applications of bis-adduct isomers of phenyl-C_{61} butyric acid methyl ester

Xueyan Hou,[†‡] Jack F. Coker,[†] Jun Yan,[†§] Xingyuan Shi,[†] Mohammed Azzouzi,[†] Flurin D. Eisner,[†] James D. McGettrick,[⁋] Sachetan M. Tuladhar,[†] Isaac Abrahams,[‡] Jarvist M. Frost,[†] Zhe Li,[⊥] T. John S. Dennis,*[‖] Jenny Nelson*[†]

[†] Department of Physics, Imperial College London, London SW7 2AZ, United Kingdom.

[‡] School of Physical and Chemical Sciences, Queen Mary University of London, London, E1 4NS, United Kingdom.

[§] School of Science and Engineering, The Chinese University of Hong Kong, Shenzhen, Guangdong Province, 518172, P. R. China

[⁋] SPECIFIC, Swansea University Bay Campus, Swansea, Wales, SA1 8EN, United Kingdom.

[⊥] School of Engineering and Materials Sciences, Queen Mary University of London, London, E1 4NS, United Kingdom.

[‖] Department of Chemistry, Xi'an Jiaotong-Liverpool University, Suzhou, 215123, China.

*Corresponding authors

John.dennis@xjtlu.edu.cn , jenny.nelson@imperial.ac.uk

Naming and Structure Assignments of Bis-PCBM Isomers

The label number of each carbon atom on C_{60} cage is shown in Figure S1. As all double bonds on C_{60} cage are symmetrically equivalent, the first addend crosses carbon atoms 1 and 9 by default. *Cis* isomers: two side chains are located at the same hemisphere; *e* isomers: the second side chain in the equator; *trans* isomers: the second side chain is located at different hemisphere.

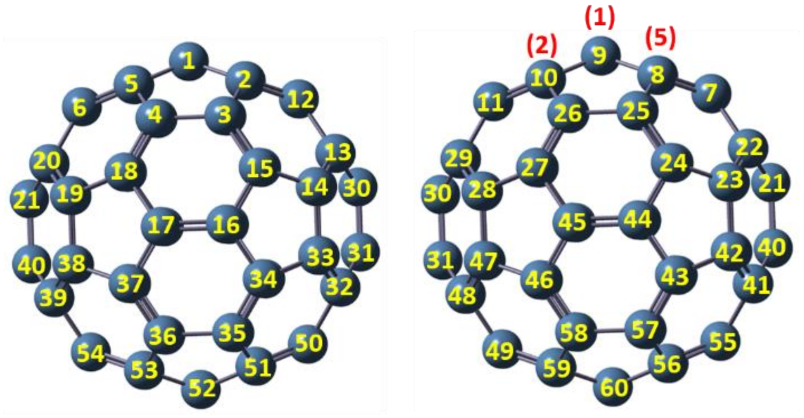

Figure S1. All carbon atom labels on C_{60} cage for the naming system of bis-PCBM isomers.

Table S1. Isomer structure assignments to the HPLC fractions with the first addend at the 1,9 position. [1] The point symmetry* of each isomer is also shown.

Bond type	HPLC fraction	Isomer assignment
trans-1	F1.1	(C_{2h}) 52,60
trans-1	F2.1.1	(C_{2v}) 52,60
trans-2	F1.2	(C_2) 49,59
trans-2	F2.1.2	(C_1) 49,59
trans-2	F3.1	(C_2) 53,54
trans-3	F2.3	(C_2) 34,35
trans-3	F3.2.1	(C_1) 34,35
trans-3	F3.3.1	(C_2) 36,37
trans-4	F2.2	(C_s) 32,33
trans-4	F3.2.2	(C_1) 32,33
trans-4	F4	(C_s) 38,39
e	F3.3.2	(C_1) 30,31
e	F5.1	(C_1) 21,40
cis-3	F3.4	(C_2) 13,14
cis-3	F5.2.1	(C_2) 19,20
cis-3	F5.2.2	(C_1) 13,14
cis-2	F5.3	(C_s) 4,18
cis-2	F6	(C_1) 3,15
cis-2	F7	(C_s) 3,15

* C_1: has no symmetry

C_2: has a single two-fold rotation axis

C_{2h}: has a two-fold rotation axis, a centre of inversion, and a horizontal mirror plane

C_{2v}: has a two-fold rotation axis and two different vertical mirror planes

C_s: has a single mirror plane

Electronic Structures

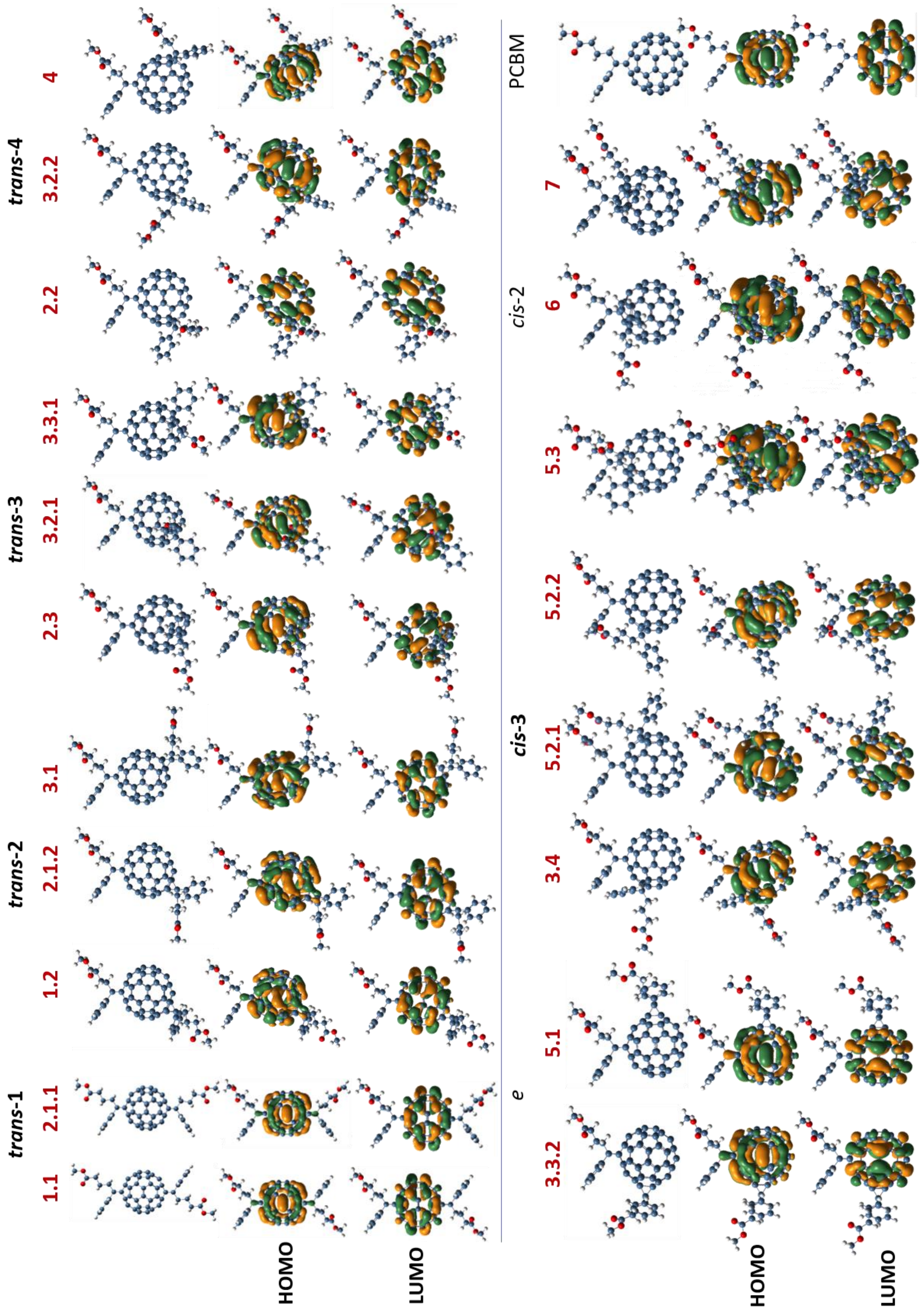

Figure S2. The molecular structures optimized using DFT (B3LYP/6-311G(2df, 2pd)) and wavefunction density distribution of the bis-PCBM isomers with the first side chain at the north pole. Isosurfaces of the HOMO and LUMO wavefunction are also exhibited.

Table S2. HOMO, LUMO and HOMO-LUMO gap energies of the bis-PCBM isomers from density functional theory calculation (T), isomer solution using cyclic voltammetry and UV-vis (S) and isomer film using UPS and UV-vis (F). The theoretical HOMO energy is calculated using DFT and the LUMO energy was estimated by adding the HOMO energy to the HOMO-LUMO gap which is the transition energy of the first singlet excitation calculated using TD-DFT using the same functional and basis set. The experimental HOMO energies are estimated from the oxidation potential of fullerenes in solution measured by CV and of thin films measured using UPS. The experimental LUMO energies are estimated, for fullerenes in solution, from the reduction potential measured using CV and, for films, from the UPS measured HOMO energy plus the thin-film energy gap measured from the UV-vis spectra.

Isomer	HOMO-T/eV	LUMO-T/eV	Gap-T/eV	LUMO-S/eV	HOMO-S/eV	Gap-S/eV	LUMO-F/eV	HOMO-F/eV	Gap-F/eV
PCBM	-5.916	-3.992	1.924	-3.917	-5.639	1.722	-4.618	-6.279	1.661
Bismix	--	--	--	-3.823	-5.528	1.705	-4.525	-6.162	1.637
F1.1	-5.713	-3.027	2.689	-	-	-	--	--	1.641
F2.1.1	-5.716	-3.030	2.683	-3.901	-5.619	1.718	--	--	1.642
F1.2	-5.732	-3.849	1.883	-3.856	-5.561	1.705	-4.637	-6.265	1.628
F2.1.2	-5.748	-3.853	1.895	-3.841	-5.553	1.712	-4.605	-6.250	1.645
F3.1	-5.766	-3.857	1.909	-3.84	-5.561	1.721	-4.533	-6.174	1.641
F2.3	-5.684	-3.796	1.888	-3.791	-5.483	1.692	-4.484	-6.103	1.619
F3.2.1	-5.701	-3.800	1.901	-3.778	-5.487	1.709	-4.462	-6.082	1.620
F3.3.1	-5.724	-3.807	1.917	-3.794	-5.511	1.717	-4.516	-6.135	1.619
F2.2	-5.670	-3.766	1.904	-3.818	-5.535	1.717	-4.473	-6.122	1.649
F3.2.2	-5.671	-3.767	1.905	-3.729	-5.444	1.715	-4.471	-6.140	1.669
F4	-5.664	-3.766	1.898	-3.842	-5.559	1.717	-4.471	-6.120	1.649
F3.3.2	-5.740	-3.566	2.174	-3.790	-5.670	1.880	-4.450	-6.190	1.740
F5.1	-5.749	-3.586	2.163	-3.790	-5.673	1.883	-4.484	-6.230	1.746
F3.4	-5.634	-3.790	1.844	-3.783	-5.447	1.664	-4.563	-6.173	1.610
F5.2.1	-5.652	-3.783	1.869	-3.785	-5.472	1.687	-4.562	-6.180	1.618
F5.2.2	-5.637	-3.784	1.853	-3.803	-5.475	1.672	-4.629	-6.240	1.611
F5.3	-5.819	-3.712	2.107	-3.782	-5.509	1.727	-4.509	-6.185	1.676
F6	-5.828	-3.695	2.133	-3.823	-5.577	1.754	-4.590	-6.270	1.680
F7	-5.756	-3.368	2.388	-3.787	-5.546	1.759	--	--	1.687

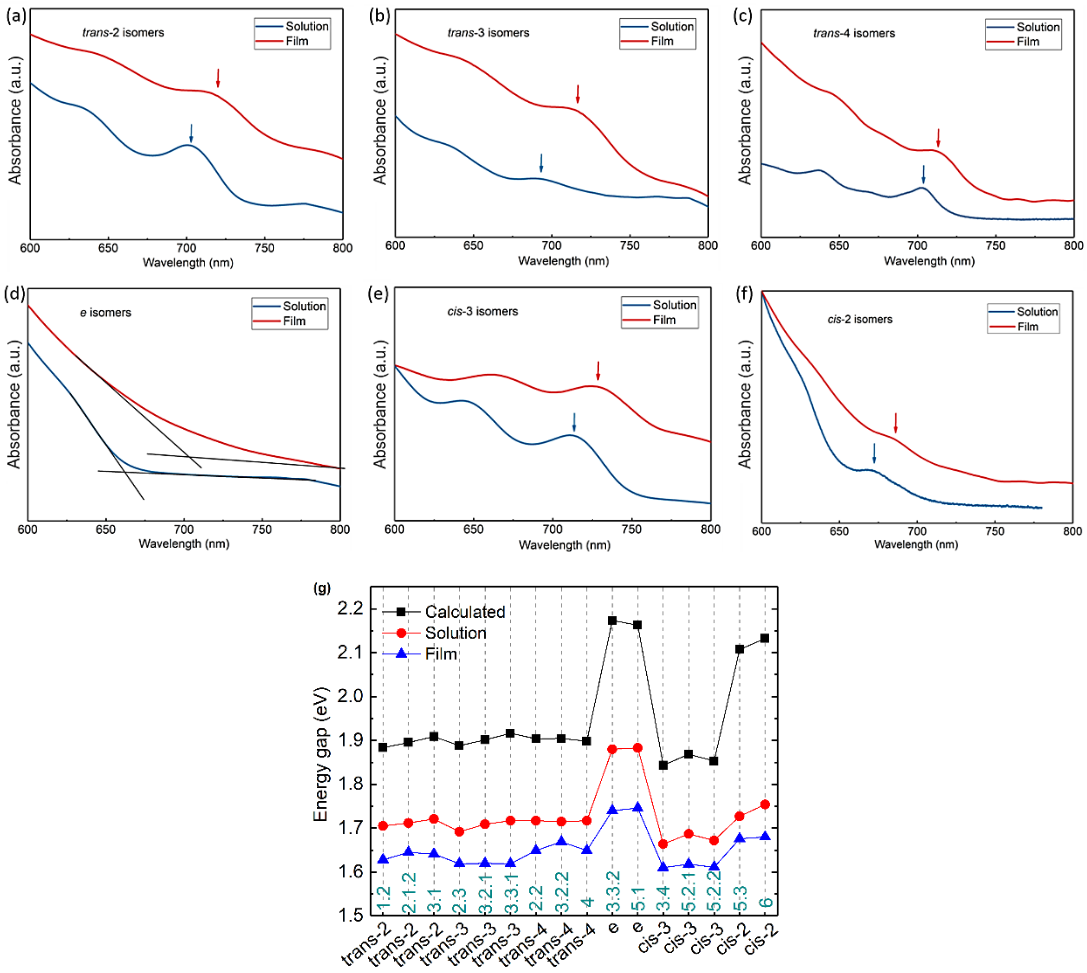

Figure S3. (a-f) The UV-vis spectra of the different types of bis-PCBM isomer in solution (toluene) and thin film.[2] (g) The comparison among the HOMO-LUMO gaps from the theoretical calculation and the UV-vis measurement of the solution and film isomers.

TGA/DSC

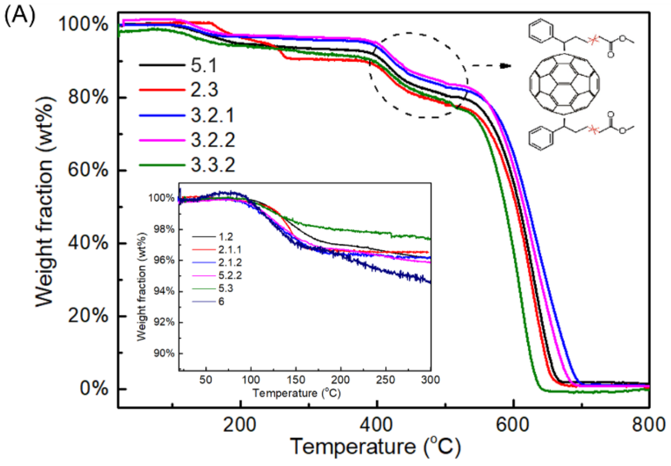

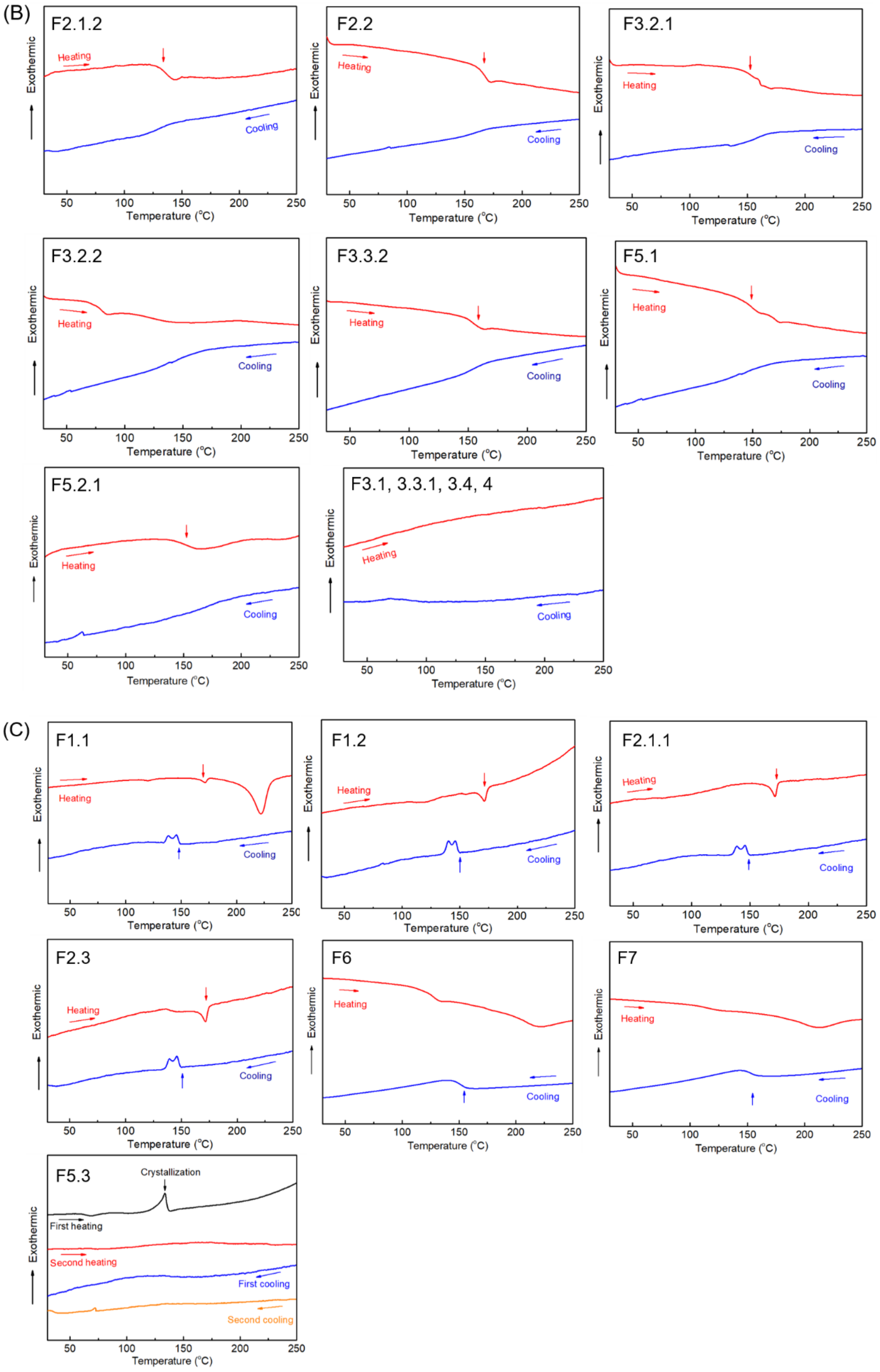
(B)
F2.1.2
F2.2
F3.2.1
F3.2.2
F3.3.2
F5.1
F5.2.1
F3.1, 3.3.1, 3.4, 4
Heating
Cooling
Exothermic
Temperature (°C)
50 100 150 200 250
(C)
F1.1
F1.2
F2.1.1
F2.3
F6
F7
F5.3
Crystallization
First heating
Second heating
First cooling
Second cooling

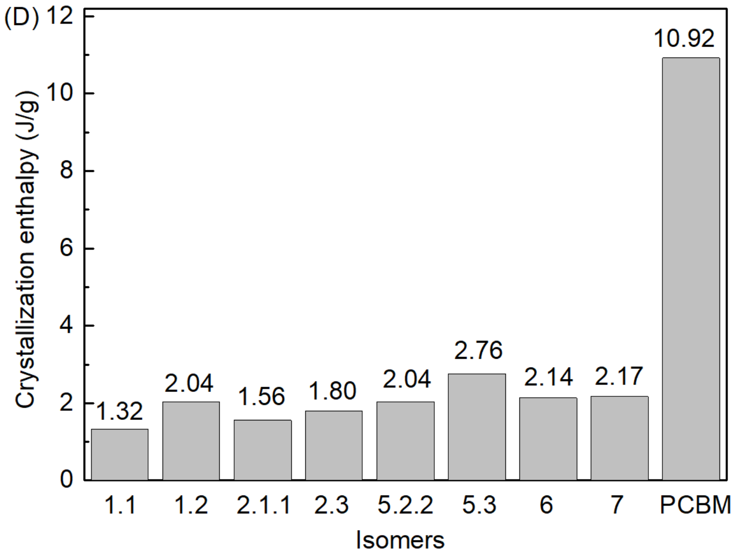

Figure S4. (A) TGA thermograms of the bis-PCBM single isomers. Some of the isomers ran TGA in the temperature range of 20 °C ~ 300 °C. (B) DSC thermograms for isomers which have no crystallization phase. (C) DSC thermograms for isomers which show crystallization peaks. All the DSC curves were from the second heating and second cooling process. (D) The crystallization enthalpy of crystalline isomers and PCBM calculated from the crystallization peak area.

FTIR/NMR

The FTIR spectra of bis-PCBM isomers were measured in air atmosphere in transmission mode using a Bruker Alpha P FTIR spectrometer with DGTS detector. The spectra were recorded from 700 to 3800 cm^{-1} with 1 cm^{-1} step size. ^1H NMR spectra were recorded on a Bruker AVNEO_600 spectrometer at 298 K using tetramethylsilane (TMS) as an internal standard. Deuterated Chloroform was used for the NMR measurements.

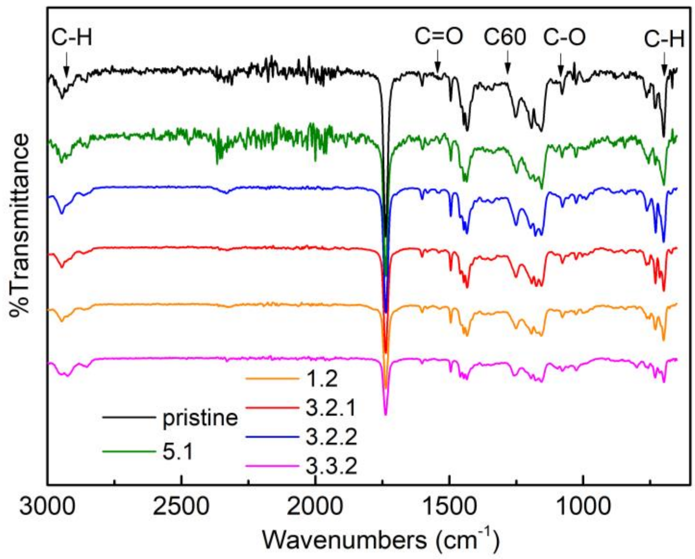

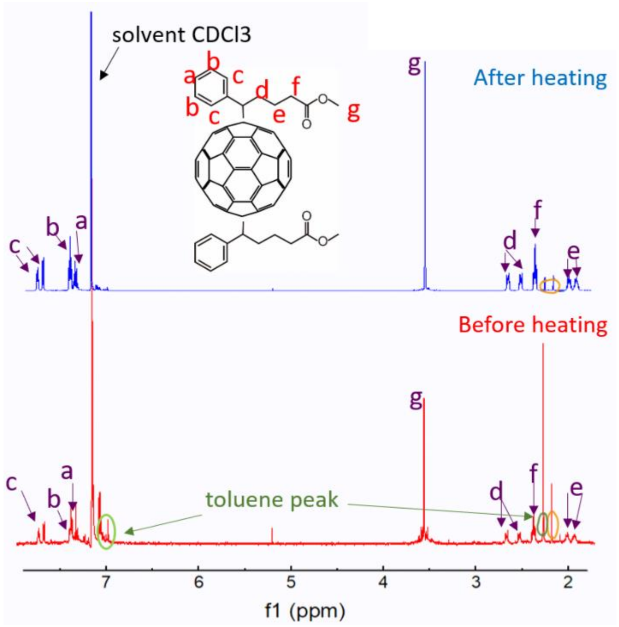

Figure S5. (a) FTIR spectra of the isomers before and after heating at 120 °C under N_2. (b) The ^1H NMR spectra of isomer 5.1 before and after heating at 120 °C. There are some grease peaks near the toluene peak.

Single Crystal X-ray Diffraction

Single crystals of isomers 2.3 and 3.3.2 were obtained using antisolvent evaporation method. [3, 4] Approximately 5 mg of sample was placed in 2 ml of IPA in a small vial and heated under constant stirring on a hotplate to 110 °C, which is between the boiling point of IPA and CB. CB (ca. 1 ml) was added dropwise along the inner vial wall until all the sample had dissolved and the solution then cooled slowly to room temperature. The cooled solution was then filtered through a 0.45 µm membrane filter into a second vial with a sealed cover. The cover was pierced with a small hole to allow slow evaporation of the solvent. After several days, crystals were found on the inner wall. At this point most of the solvent mixture was removed using a teat pipette and the crystals washed with IPA. The crystals were then transferred onto a silicon slide for characterization. Crystals for isomer 7 were easy to obtain, and were precipitated from toluene solution directly without an antisolvent.

Single-crystal X-ray diffraction data were collected at the UK National Crystallography Service at the University of Southampton on a Rigaku, 007-HF 4-circle diffractometer fitted with a Rigaku, HyPix 6000 hybrid pixel detector. Data were collected at 100 K using Cu-Kα radiation (λ = 1.5418 Å). The structures were solved with using direct methods in SHELXTL [5] and refined within SHElXL [6] within the WINGX Gui. [7] Molecular graphics and C_{60} centroid- C_{60} centroid distances were obtained using CrystalMaker. [8]

Figure S6 shows the X-ray single crystal structure of isomers 2.3, 3.3.2 and 7 with thermal ellipsoids representing 50% probability.

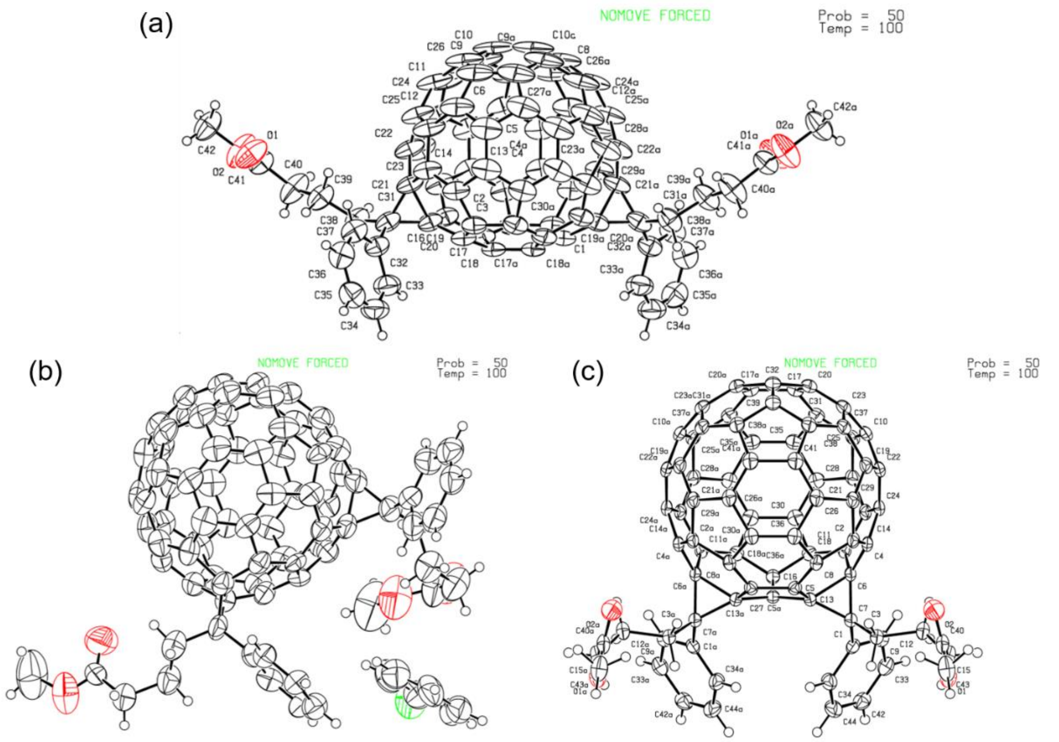

Figure S6. X-ray single-crystal structure of (a) isomer 2.3, (b) isomer 3.3.2, (c) isomer-7 in vacuum. The solvent molecules in isomer 2.3 and isomer 7 are omitted for clarity.

Solar Cell Performance

Table S3. Extracted device parameters from current-voltage characteristics of ITO/PEDOT:PSS(30 nm)/P3HT:fullerene(1:1, 200 nm)/Ca(20 nm)/Al(80 nm) devices. The errors were calculated from at least 12 cells. The PCE value given in brackets is the highest single value measured. The condition 'crystalline' refers to whether the fullerene showing a crystalline peak in DSC.

Fullerene	Crystalline (Yes/No)	J_{SC} (mA/cm^2)	V_{OC} (V)	FF (%)	PCE (%) (max)
PCBM	Y	6.88±0.30	0.6±0.004	60.5±2.6	2.67±0.23 (3.01)
Bis mix	N	5.40±0.29	0.734±0.006	55.5±1	2.19±0.14 (2.44)
1.2, *trans*-2	Y	5.77±0.50	0.725±0.005	55±1.4	2.30±0.23 (2.74)
2.1.2, *trans*-2	N	6.84±0.39	0.759±0.005	56.1±1.1	2.92±0.17 (3.26)
3.2.1, *trans*-3	N	6.36±0.24	0.77±0.004(0.78)	57.6±2.8	2.82±0.18 (3.10)
2.2, *trans*-4	N	6.33±0.29	0.77±0	61.4±1	2.99±0.11 (3.12)
3.2.2, *trans*-4	N	6.13±0.39	0.766±0.007	60±1.7	2.82±0.17 (3.12)
3.3.2, *e*	N	6.40±0.18	0.757±0.006	61.7±1.2	2.96±0.13 (3.15)
5.1, *e*	N	6.77±0.25	0.764±0.006	61.3±1.5	3.16±0.13 (3.38)
5.2.2, *cis*-3	Y	5.62±0.59	0.708±0.004	54.4±1.7	2.23±0.21 (2.55)
6, *cis*-2	Y	6.21±0.3	0.712±0.004	54.6±1.6	2.44±0.16 (2.72)

Table S4. Extracted device parameters from current-voltage characteristics of ITO/PEDOT:PSS(30 nm)/PCDTBT:fullerene(1:2, 85 nm)/PFN(5 nm)/Al(100 nm) and ITO/PEDOT:PSS(30 nm)/PBDB-T:fullerene(1:1, 85 nm)/ PFN(5 nm)/Al(100 nm) devices.

Active layer	***crystalline***	V_{OC} [V]	J_{SC} [mA/cm^2]	FF [%]	PCE [%]
PCDTBT:PCBM	Y	0.93±0	8.81±0.21	62±1.3	5.15±0.14 (5.4)
PCDTBT:bis-mixture	N	1.04±0.004	6.42±0.24	41±1	2.78±0.1 (2.88)
PCDTBT:3.2.1-*trans*-3	N	1.09±0.005	7.42±0.15	47±0.86	3.87±0.08 (4.01)
PCDTBT:3.2.2-*trans*-4	N	1.06±0.005	7.18±0.24	45±1.1	3.50±0.11 (3.70)
PCDTBT:5.1-*e*	N	1.05±0.005	8.17±0.22	51±1.23	4.45±0.12 (4.62)
PCDTBT:5.2.2-*cis*-3	Y	1.02±0.004	6.53±0.19	40±1.31	2.60±0.13 (2.75)
PBDB-T:PCBM	Y	0.84±0.004	12.30±0.30	63±1.26	6.82±0.21 (7.03)
PBDB-T:bis-mixture	N	0.95±0.006	9.54±0.28	52±1.3	5.00±0.15 (5.21)
PBDB-T: 3.2.1-*trans*-3	N	0.97±0.005	11.31±0.23	57±1.29	6.10±0.17 (6.31)

PBDB-T:3.2.2-*trans*-4	N	0.97±0.004	10.53±0.19	56±1.27	5.90±0.15 (6.05)
PBDB-T:5.1-*e*	N	0.96±0.005	11.95±0.13	61±0.96	7.00±0.15 (7.2)
PBDB-T:5.2.2-*cis*-3	Y	0.94±0.006	8.48±0.16	49±1.31	4.52±0.18 (4.71)

Voltage Loss Analysis

The voltage losses were investigated quantitatively using electroluminescnce (EL) measurements along with sub-band-gap external quantum efficiency (EQE) measurements. Four quantities can be determined according to the specified loss mechanisms: 1) $V_{oc,sq}$, the maximum open-circuit voltage of a device with the band gap of the studied device, operating in the radiative (Shockly-Quiesser) limit; 2) $V_{oc,rad}$, the open-circuit voltage of the studied device when operating in the radiative limit; 3) $\Delta V_{oc,abs}$, the difference between $V_{oc,sq}$ and $V_{oc,rad}$, which can be assigned to the shape of the EQE spectrum edge; 4) $\Delta V_{oc,nr}$, the difference between $V_{oc,rad}$ and V_{oc} representing the voltage loss due to non-radiative recombination.[9-11]

The normalized EL and EQE spectra for the P3HT:fullerene devices is shown in Figure S7. The EQE are composed of directly measured quantum efficiency and the quantum efficiency determined from the EL spectra, from which the radiative recombination $V_{oc,rad}$ can be calculated based on the method described in detail by Yao et al.[9] Then the $\Delta V_{oc,abs}$ and $\Delta V_{oc,nr}$ values can be calculated by $V_{oc,sq}$ - $V_{oc,rad}$ and $V_{oc,rad}$ - V_{oc} .

The EQE spectra were measured using a grating spectrometer (CS260-RG-4-MT-D) to create monochromatic light combined with a tungsten halogen light source. The monochromatic light was chopped at 300 Hz, and a Stanford Research System SR380 lock-in amplifier with an internal transimpedance amplifier of 10^6 V/A was used to detect the photocurrent. Long pass filters at 610, 715, 780, 850, and 1000 nm were used to filter out the scattered light from the monochromator. The spectra were taken from 300 to 1100 nm and calibrated by a silicon photodiode.

EL was measured using a Shamrock 303 spectrograph combined with an iDUS InGaAs array detector cooled to -90 °C. The obtained EL spectra intensity was calibrated with the spectrum from a calibrated halogen lamp and the raw spectrum was corrected by subtracting a dark spectrum and using a calibration file that corrects for the detector sensitivity at different wavelengths. A Keithley 2450 source meter was used to provide power to drive the samples.

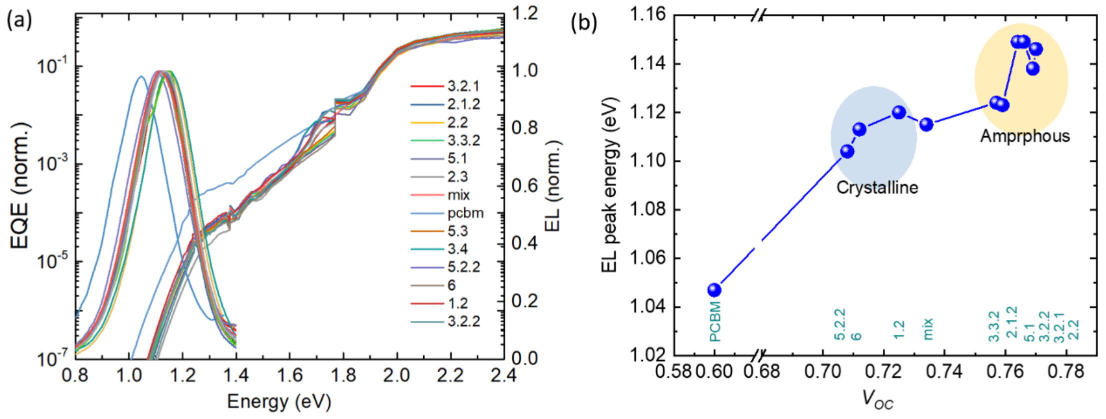

Figure S7. (a) Normalized EL and EQE spectra for the P3HT:fullerene devices, (b) EL peak energy as a function of open circuit voltage. The EQE are composed of directly measured quantum efficiency and the quantum efficiency determined from the EL spectra.

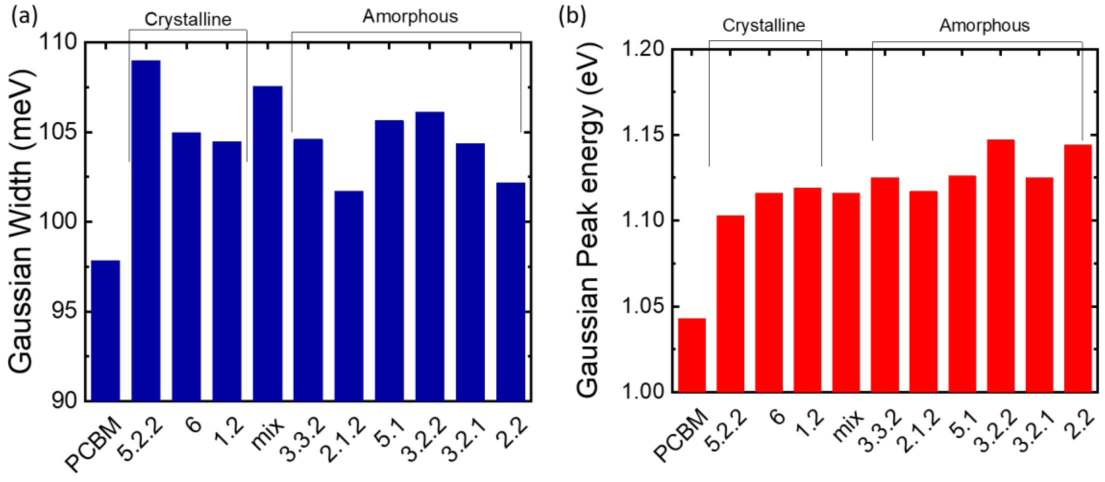

Figure S8. (a) Extracted gaussian width of the EL spectra and (b) peak EL energy for organic photovoltaic devices based on P3HT polymer blended with different bis-isomers.

(A)

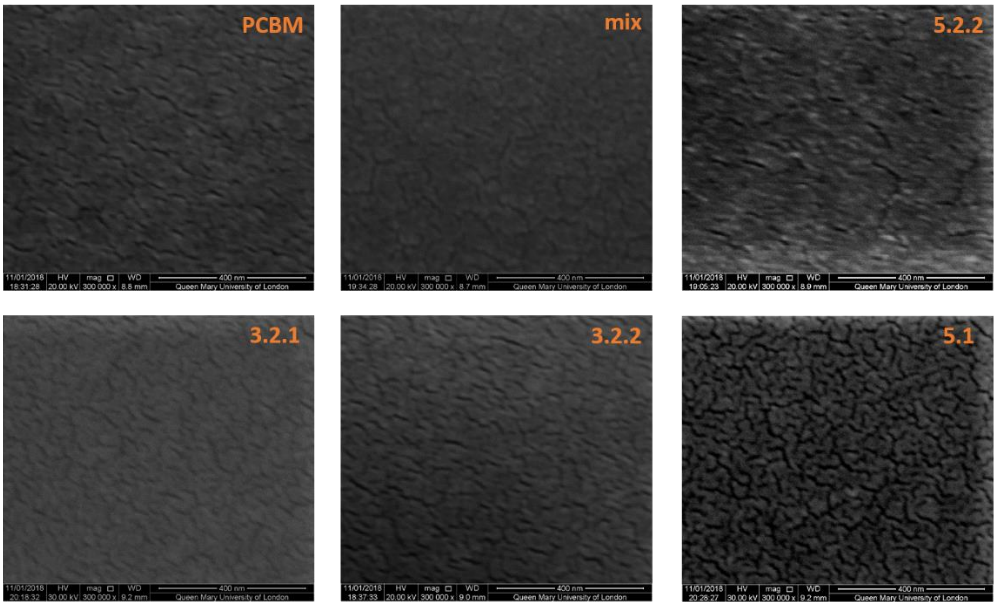

(B)

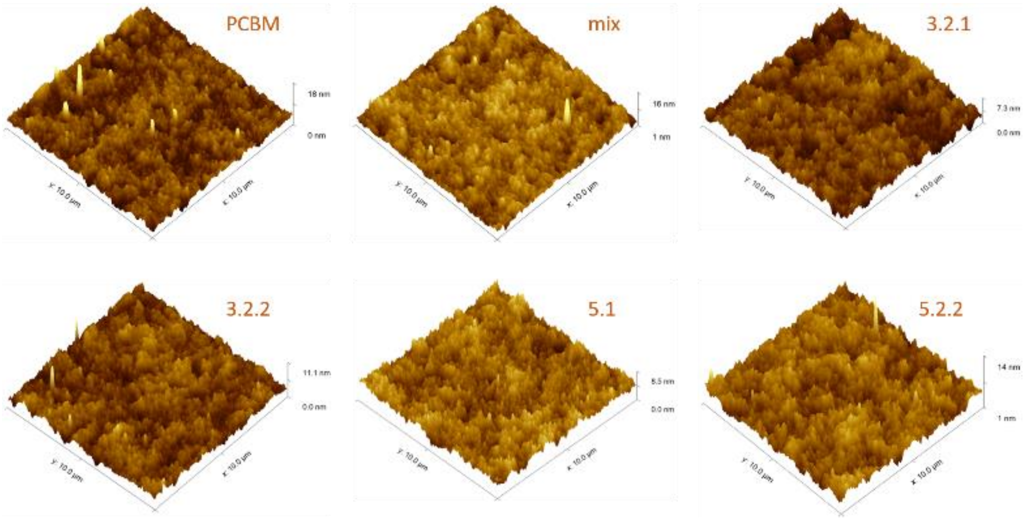

Figure S9. (A) The SEM images of P3HT:fullerene blend films (on ITO/PEDOT:PSS) made using PCBM, the bis-PCBM mixture and individual isomers as named. The scale bar is 400 nm. SEM images were recorded on an FEI Inspect-F scanning electron microscope. (B) The AFM images of the PBDB-T:fullerene blend films, exhibiting amorphous and smooth property.[12] The blends with PCBM, bis-mix and crystalline isomer-5.2.2 show slightly higher roughness of 18 nm, 16 nm, and 14 nm. The blends with amorphous isomers 3.2.1, 3.2.2 and 5.1 show a smaller roughness of 7.3 nm, 8.5 nm, and 11.1 nm.

Charge Transport Simulations

Model System

For each of the crystal structures studied, the centroid positions of the C_{60} cages were first found within the unit cell. These are referred to as sites. 3×3×3 supercells was constructed using these site positions. Pairwise distances, r_{ij}, between sites were computed using a function from the python package MDAnalysis.[13, 14] This function handles the periodic boundaries of the supercell by applying the minimum image convention. The electron transfer integrals between molecule pairs were estimated using

$$J_{ij}(r_{ij}) = \alpha \exp\left(-r_{ij}/\beta\right) \tag{1}$$

Values for the parameters α and β were chosen following previous work by Steiner and colleagues and are given in Table S5.[15] Because the charge transport simulation program assumes an external electric field oriented along the *z*-axis, using the unmodified site coordinates yields the mobility in the $a\times b$ direction of the crystal. To study transport in directions $b\times c$ and $c\times a$, the site positions were rotated around the origin such that the *b*-*c* and *c*-*a* crystal planes were parallel with the *x*-*y* plane.

Table S5. Input parameters for charge transport simulations

**Parameters**	**Symbols**	**Values**	**Units**
Temperature	T	300	K
External electric field strength	F	1×10^4	V cm^{-1}
Transfer integral prefactor	α	15	MeV
Transfer integral decay constant	β	0.5	Å
Reorganisation Energy (Inner-sphere only)	λ_i	0.2	eV
Reorganisation Energy (Total)	λ	0.5	eV

Charge Transport Simulation

Mobilities were found by solving the steady state Master Equation for the system:

$$\sum_{i\neq j}\left[\Gamma_{i\to j}P_i - \Gamma_{j\to i}P_j\right] = 0 \tag{2}$$

which can be expressed in matrix form as

$$\boldsymbol{A} \cdot \boldsymbol{P} = \boldsymbol{0}, \quad with \quad A_{mn} = \begin{cases} \Gamma_{i\to j}, & i \neq j \\ -\sum_{i\neq j} \Gamma_{j\to i}, & i = j \end{cases} . (3)$$

Where $\Gamma_{i\to j}$ is the rate constant at which charges hop from site i to site j, and P_i is the occupation probability of site i and it is assumed that all P_i are close to 0. Hopping rates were computed using semi-classical Marcus theory:

$$\Gamma_{i\to j} = \frac{|J_{ij}|^2}{\hbar} \sqrt{\frac{\pi}{\lambda k_B T}} \exp\left(-\frac{(\Delta E_{i\to j} + \lambda)^2}{4\lambda k_B T}\right). (4)$$

The term $\Delta E_{i\to j}$ describes the difference in energy between sites i and j, $\Delta E_{i\to j} = E_j - E_i$ where E_i is the energy of an electron in site i. In the absence of energetic disorder, this difference arises solely due to the presence of the external electric field, which modifies $\Delta E_{i\to j}$ like:

$$\Delta E_{i\to j} = -eF\vec{r}_{ij} \cdot \hat{z}, (5)$$

where the negative sign indicates we are studying electron transport. The system of simultaneous equations described by Equation 3 was solved by singular value decomposition, using a function from the GNU Scientific Library.[16] This yields occupation probabilities of the sites, $\boldsymbol{P}$. Using these, we can compute the drift velocity of the charges according to

$$v = \sum_{i\neq j} A_{ij} P_j \vec{r}_{ij} \cdot \hat{z}, (6)$$

from which the mobility is found using

$$\mu = \frac{v}{F}. (7)$$

Table S6. Electron transport mobilities computed using total reorganisation energy of 0.5 eV, i.e. external contribution of 0.3 eV. Unit for mobility is cm^2 V^{-1} s^{-1}.

Field Direction	Crystal 2.3	Crystal 3.3.2	Crystal 7
$b \times c$	0.10	0.03	0.07
$c \times a$	0.10	0.04	1×10^{-6}
$a \times b$	4×10^{-11}	2×10^{-7}	0.01

References

1. Liu, T.; Abrahams, I.; Dennis, T. J. S., Structural Identification of 19 Purified Isomers of the OPV Acceptor Material bisPCBM by 13C NMR and UV–Vis Absorption Spectroscopy and High-Performance Liquid Chromatography. *The Journal of Physical Chemistry A* **2018,** *122* (16), 4138-4152.
2. Shi, W.; Hou, X.; Liu, T.; Zhao, X.; Sieval, A. B.; Hummelen, J. C.; Dennis, T. J. S., Purification and electronic characterisation of 18 isomers of the OPV acceptor material bis-[60] PCBM. *Chemical Communications* **2017,** *53* (5), 975-978.

3. Zhao, X.; Liu, T.; Cui, Y.; Hou, X.; Liu, Z.; Dai, X.; Kong, J.; Shi, W.; Dennis, T. J. S., Antisolvent-assisted controllable growth of fullerene single crystal microwires for organic field effect transistors and photodetectors. *Nanoscale* **2018,** *10* (17), 8170-8179.
4. Zhao, X.; Liu, T.; Hou, X.; Liu, Z.; Shi, W.; Dennis, T. J. S., [60] PCBM single crystals: remarkably enhanced band-like charge transport, broadband UV-visible-NIR photo-responsivity and improved long-term air-stability. *Journal of Materials Chemistry C* **2018,** *6* (20), 5489-5496.
5. Sheldrick, G. M., SHELXT–Integrated space-group and crystal-structure determination. *Acta Crystallographica Section A: Foundations and Advances* **2015,** *71* (1), 3-8.
6. Sheldrick, G. M., Crystal structure refinement with SHELXL. *Acta Crystallographica Section C: Structural Chemistry* **2015,** *71* (1), 3-8.
7. Farrugia, L. J., WinGX suite for small-molecule single-crystal crystallography. *Journal of Applied Crystallography* **1999,** *32* (4), 837-838.
8. Palmer, D. C., Visualization and analysis of crystal structures using CrystalMaker software. *Zeitschrift für Kristallographie-Crystalline Materials* **2015,** *230* (9-10), 559-572.
9. Yao, J.; Kirchartz, T.; Vezie, M. S.; Faist, M. A.; Gong, W.; He, Z.; Wu, H.; Troughton, J.; Watson, T.; Bryant, D.; Nelson, J., Quantifying losses in open-circuit voltage in solution-processable solar cells. *Physical review applied* **2015,** *4* (1), 014020.
10. Street, R. A.; Hawks, S. A.; Khlyabich, P. P.; Li, G.; Schwartz, B. J.; Thompson, B. C.; Yang, Y., Electronic Structure and Transition Energies in Polymer–Fullerene Bulk Heterojunctions. *The Journal of Physical Chemistry C* **2014,** *118* (38), 21873-21883.
11. Azzouzi, M.; Yan, J.; Kirchartz, T.; Liu, K.; Wang, J.; Wu, H.; Nelson, J., Nonradiative energy losses in bulk-heterojunction organic photovoltaics. *Physical Review X* **2018,** *8* (3), 031055.
12. Hou, X.; Clarke, A. J.; Azzouzi, M.; Yan, J.; Eisner, F.; Shi, X.; Wyatt, M. F.; Dennis, T. J. S.; Li, Z.; Nelson, J., Relationship between molecular properties and degradation mechanisms of organic solar cells based on bis-adducts of phenyl-C61 butyric acid methyl ester. *Journal of Materials Chemistry C* **2022,** *10*, 7875-7885.
13. Gowers, R. J.; Linke, M.; Barnoud, J.; Reddy, T. J. E.; Melo, M. N.; Seyler, S. L.; Domanski, J.; Dotson, D. L.; Buchoux, S.; Kenney, I. M.; Beckstein, O., MDAnalysis: A Python Package for the Rapid Analysis of Molecular Dynamics Simulations. In *Conference: PROC. OF THE 15th PYTHON IN SCIENCE CONF. (SCIPY 2016) ; 2016-07-11 - 2016-07-11 ;*, United States, 2019; p Medium: ED; Size: 98.
14. Michaud-Agrawal, N.; Denning, E. J.; Woolf, T. B.; Beckstein, O., MDAnalysis: A toolkit for the analysis of molecular dynamics simulations. *Journal of Computational Chemistry* **2011,** *32* (10), 2319-2327.
15. Steiner, F.; Foster, S.; Losquin, A.; Labram, J.; Anthopoulos, T. D.; Frost, J. M.; Nelson, J., Distinguishing the influence of structural and energetic disorder on electron transport in fullerene multi-adducts. *Materials Horizons* **2015,** *2* (1), 113-119.
16. al, M. G. e., *GNU Scientific Library Reference Manual (3rd Ed.), ISBN 0954612078.* 2019.
